# Molecular Mapping of Putative Genomic Regions Controlling Fruit and Seed Morphology of Watermelon

**DOI:** 10.3390/ijms242115755

**Published:** 2023-10-30

**Authors:** Tiantian Yang, Sikandar Amanullah, Shenglong Li, Rui Cheng, Chen Zhang, Zhengxiang Zhao, Hongyu Liu, Feishi Luan, Xuezheng Wang

**Affiliations:** 1 College of Horticulture and Landscape Architecture, Northeast Agricultural University, Harbin 150030, China; yttneau2021@gmail.com (T.Y.); amahakurohane@163.com (S.L.); cheng.rui@foxmail.com (R.C.); zzxneau@163.com (Z.Z.); hyliu@neau.edu.cn (H.L.); luanfeishi@neau.edu.cn (F.L.); 2 Key Laboratory of Biology and Genetic Improvement of Horticulture Crops (Northeast Region), Ministry of Agriculture and Rural Affairs, Harbin 150030, China

**Keywords:** ABC transporter, CAPS markers, candidate gene, linkage mapping, QTL

## Abstract

The genetic regulatory basis of qualitative and quantitative phenotypes of watermelon is being investigated in different types of molecular and genetic breeding studies around the world. In this study, biparental F_2_ mapping populations were developed over two experimental years, and the collected datasets of fruit and seed traits exhibited highly significant correlations. Whole-genome resequencing of comparative parental lines was performed and detected single nucleotide polymorphism (SNP) loci were converted into cleaved amplified polymorphic sequence (CAPS) markers. The screened polymorphic markers were genotyped in segregating populations and two genetic linkage maps were constructed, which covered a total of 2834.28 and 2721.45 centimorgan (cM) genetic lengths, respectively. A total of 22 quantitative trait loci (QTLs) for seven phenotypic traits were mapped; among them, five stable and major-effect QTLs (PC-8-1, SL-9-1, SWi-9-1, SSi-9-1, and SW-6-1) and four minor-effect QTLs (PC-2-1 and PC-2-2; PT-2-1 and PT-2-2; SL-6-1 and SSi-6-2; and SWi-6-1 and SWi-6-2) were observed with 3.77–38.98% PVE. The adjacent QTL markers showed a good fit marker-trait association, and a significant allele-specific contribution was also noticed for genetic inheritance of traits. Further, a total of four candidate genes (*Cla97C09G179150*, *Cla97C09G179350*, *Cla97C09G180040*, and *Cla97C09G180100*) were spotted in the stable colocalized QTLs of seed size linked traits (SL-9-1 and SWi-9-1) that showed non-synonymous type mutations. The gene expression trends indicated that the seed morphology had been formed in the early developmental stage and showed the genetic regulation of seed shape formation. Hence, we think that our identified QTLs and genes would provide powerful genetic insights for marker-assisted breeding aimed at improving the quality traits of watermelon.

## 1. Introduction

Watermelon is a popular fruit that belongs to the Cucurbitaceae family. It has become an attractive model fruit for studying the key biological mechanisms that control multivariate phenotypes [1,2,3]. Numerous genetic breeding approaches enabled the development of cultivated varieties of watermelons with improved traits [4]; however, after a long time of artificial domestication and selection, the genetic background of cultivated watermelons is much narrower than that of the original wild varieties [5]. Although the improved reference genome has been published, the stable genomic regions and key genes regulating complex traits are still being investigated in many unexplored botanical groups of watermelon [4,5].

Fruit weight and peel thickness are complex but typical quantitative traits of watermelon [6]. QTLs affecting the single fruit weight of watermelon were pinpointed on different chromosomes (2, 3, 6, 8, 9, and 11); among which, fwt9.2 QTL was the most dominant QTL positioned on chromosome 9, contributing 45.70% of the genetic effects of the variations [1,7,8]. For the peel thickness, QTLs were mainly identified on chromosomes 2, 3, 6, 7, 9, and 10, and rth9.1 QTL positioned on chromosome 9 depicted dominant genetic effects. Recently, a genome-wide association study (GWAS) uncovered valuable insights into the natural variation and phenotypic correlations in peel thickness and fruit weight of watermelon [9], indicating the shared genetic makeup of these traits [1,7,10,11]. Fruit and seed size are important factors for both consumers and breeding companies [12]. In earlier domestication, sweet watermelons were known with thin peels and small fruit and seed sizes, while the latter pursued higher profits and preferred products with big fruits, thick peels, and large seeds. Therefore, it is of great significance to identify the candidate QTLs of commercial traits aimed at breeding varieties that meet the needs of consumer diversity.

Fruit peel color also ranges from light to dark green [8,12]. The basic genetic model of green peel was proposed for the first time and stated that the dark green peel had more genetic effects than the light green peel, which is regulated by multiple major and minor genes and interferes with the synthesis of peel color in watermelon [13]. This inheritance model was also identified as consistent with identical dominant epistasis, in which two allele pairs jointly regulate the dark-green phenotype [14]. Later, multiple genetic basis for peel color were dissected in the divergent cultivars of watermelon. Genetic linkage mapping exposed a candidate genetic marker for the yellow skin color of watermelon [15]. A single genetic locus harboring the *ClCG08G017810* gene encoding the 2-phytyl-1,4-beta-naphthoquinone methyltransferase protein was proposed, and gene expression levels in the dark green peel were noticed to be significantly higher compared to the light green [16]. It was also found that the multi-allelic *APRR2* gene significantly accumulated fruit pigmentation in melon and watermelon [17]. A recently developed hybrid generation with intermediate peel colors showed that peel color inheritance depends on whether a pair of alleles is homozygous or not [8,12,18]. All these experiments have differentiated the diverse genetic backgrounds of different botanical groups of watermelon, and the genes controlling peel color still need to be further explored.

Seed is a primary component of the plant life cycle that serves to propagate [12]. The size, weight, and thickness of watermelon seed exhibit a lot of variations due to natural and artificial adaptation to different localities [12,19,20]. It was reported that seed size is governed by non-allelic genes (*l* and *s*), and a good-fit Mendelian genetic segregation was seen. Initially, a genetic model of llS as a large seed, “LS” as an intermediate seed, and “ss” as a small seed was proposed [21]. However, the segregated mapping population showed continuous variations in seed length, width, and 1000-seed weight, which may be due to different genetic effects of QTL [8]. A total of 20 QTLs of seed length, 19 QTLs of seed width, and 10 QTLs of 1000-seed weight were reported on different chromosomes (1, 2, 3, 5, 6, 8, and 11), and *ClSS6.1* and *ClSS6.2* on chromosome 6 were found to be relatively stable, exhibiting the main genetic effects of wild and cultivated materials [19]. QTL (*qSS6*) positioned on chromosome 6, explained the significant phenotypic variation effects (>90%) for seed length, seed width, and 1000-seed weight. Also, three candidate genes (*Cla009291*, *Cla009301,* and *Cla009310*) were published, including a seed-size regulator “*SRS3*” in rice [19]. It can be speculated that potential homologous genes controlling fruit and seed size are highly conserved in structure and function, which may indicate that the QTL or gene controlling fruit and seed size share the same genetic phenomenon [19]. A recent GWAS-based analysis revealed significant genetic insights about the biological transformation of watermelon seed size [6]. However, the common genetic interval across different types of research should be thought of as the candidate interval of the hypothesis, which needs to be tested by in-depth analysis.

The rapid molecular sequencing approaches and improved reference genome assemblies of watermelon have been introduced [5,22,23,24], which assisted in identifying the base-by-base single nucleotide polymorphisms (SNPs), polymorphic markers, linkage mapping, and key genes underlying candidate genomic regions [8,12]. In the recent decade, whole genome re-sequencing (WGRS)-based SNP-CAPS markers effectively assisted the primary and fine genetic mapping analysis of quantitative and qualitative phenotypes in different segregating populations developed from unexplored botanical varieties [8,11,12,25,26,27,28,29]. So, the present study was also aimed at developing and genotyping the new whole genome CAPS markers in two segregating biparental (F_2_) mapping populations for analyzing the stable QTLs conferring watermelon fruit and seed traits over two experimental years. We think that our identified QTL results will serve as an additional reliable reference for future fine genetic mapping and gene mining studies.

## 2. Results

### 2.1. Analysis of Genetic Characteristics of Phenotypes

#### 2.1.1. Peel Color (PC, Scoring)

**Figure 1:**
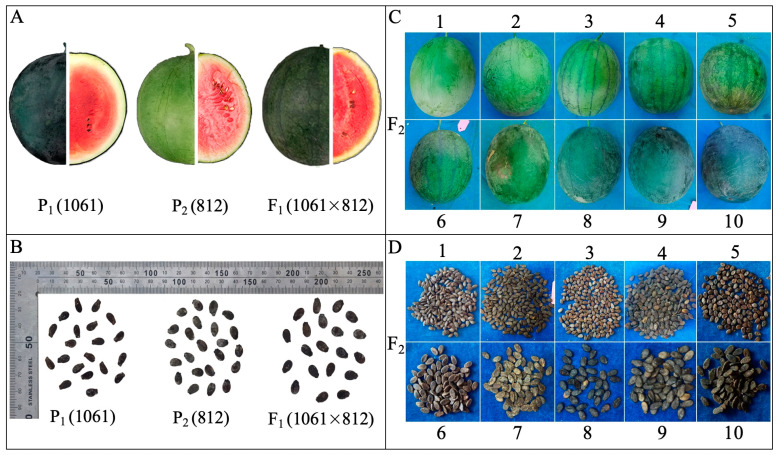
The pictorial view of primary differences in fruit and seed-linked morphological traits. (**A**) Fruit morphology of inbred parental lines (P_1_ and P_2_) and hybrid material (F_1_), (**B**) Seed morphology of inbred parental lines and F_1_ hybrid material, (**C**) Peel color differentiation, and (**D**) Seed morphology differentiation in the biparental (F_2_) mapping populations, respectively.

**Figure 2:**
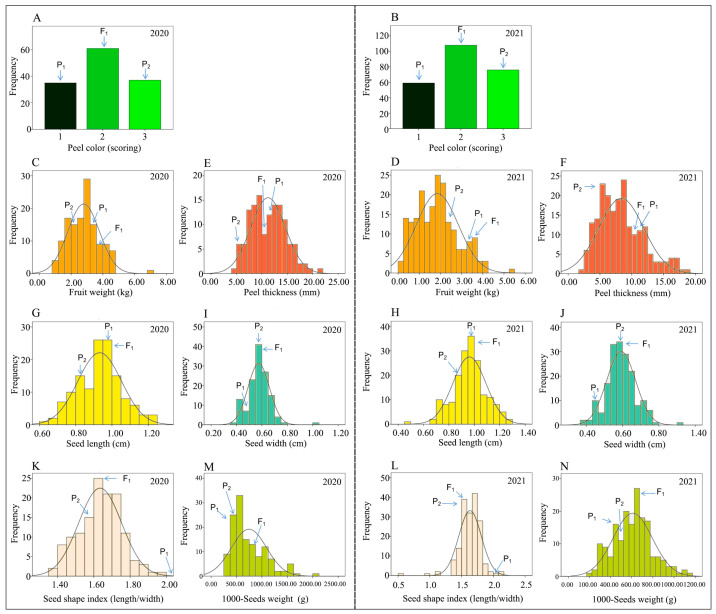
Frequency graphs of observed phenotypic traits of developed biparental (F_2_) mapping populations over two experimental years (2020 and 2021). (**A**,**B**) Peel color, (**C**,**D**) Fruit weight, (**E**,**F**) Peel thickness, (**G**,**H**) Seed length, (**I**,**J**) Seed width, (**K**,**L**) Seed shape index, and (**M**,**N**) 1000-Seed weight, respectively. P_1_ is female parent line (1061), P_2_ is male parent line (812), and F_1_ is hybrid material (1061 × 812).

The peel color of two parent lines and F_1_ was distinctive as “dark green, light green, and an intermediate color” (Figure 1A,C). In both experimental years (2020 and 2021), most of the fruits of F_2_ mapping populations exhibited obvious intermediate peel color (Figure 2A,B). The inheritance of peel color was noticed according to the genetic patterns in the parental genotypes “P_1_ (homozygous as dark green), P_2_ (homozygous as light green), and F_1_ (heterozygous as intermediate)”, which indicated that the dark green of watermelon peel was partially dominant compared to the light green, exhibiting polygenic genetics. For the observed fruits of the 2020-F_2_ mapping population, 37 fruits were dark green, 35 fruits were light green, and 61 fruits were intermediate green, and they exhibited the good-fit Mendelian segregation ratio (1:2:1) with χ^2^ = 0.33 and *p*-value = 0.15; however, the observed fruits of the 2021-F_2_ mapping population depicted a total of 76 fruits with dark green rind, 62 fruits with light green rind, and 112 fruits with intermediate green, and also exhibited the 1:2:1 segregation ratio at χ^2^ = 0.85 and *p*-value = 0.12 (Supplementary Table S1).

#### 2.1.2. Fruit Weight (FW, kg)

There were significant differences in the single fruit weight of watermelon among the comparative parents and F_1_ hybrid (3.45 ± 0.23, 2.65 ± 0.06, and 3.74 ± 0.24). The FW mean value of the 2020-F_2_ mapping population was 2.79 ± 0.99, with variations ranging from 0.90 to 7.18 kg, and the FW mean value of the 2021-F_2_ mapping population was 1.85 ± 0.99, with variations ranging from 0.12 to 5.26 kg (Table 1). In both experimental years (2020 and 2021), the main genetic variation effect was observed with medium- to heavy-weighted fruits (Figure 1C), and a continuous distribution model for FW of F_2_ population was observed (Figure 2C,D). Interestingly, the FW of F_2_ population fruits grown in 2021 was significantly shifted towards small fruit with less fruit weight as compared to the fruit weight of F_2_ population fruits developed in 2020. It was assumed that fruit weight is a multifaceted quantitative trait that is synchronized by polygenes.

#### 2.1.3. Peel Thickness (PT, mm)

There were significant differences in fruit peel thickness (mm) between P_1_ (11.44 ± 0.02), P_2_ (5.89 ± 0.11), and F_1_ hybrid (10.64 ± 0.03) (Figure 1A). The PT mean value of the 2020-F_2_ mapping population was 11.21 ± 3.42 and variations ranged from 4.73 to 21.13 mm; the PT mean value of the 2021-F_2_ mapping population was 8.33 ± 3.53 but variations ranged from 2.26 to 18.35 mm, respectively (Table 1). A normal quantitative frequency distribution of PT was observed in the harvested fruits of developed F_2_ mapping populations in both experimental years (2020 and 2021) (Figure 2E,F). Most of the dissected fruits of the two-year experimental F_2_ mapping population apparently showed more thickness of peel, exhibiting a major dominance of the female parent line and the F_1_ hybrid.

#### 2.1.4. Seed Length (SL, mm)

The average seed length (mm) of P_1_ (9.52 ± 0.07), P_2_ (8.90 ± 0.08), and F_1_ (9.620.14) significantly differed (Figure 1B). The SL mean value of the 2020-F_2_ plants was 9.32 ± 1.20, and low to high variations were noticed from 6.40 to 12.30 mm; however, the SL mean value of the 2021-F_2_ mapping population was 9.61 ± 1.30, but variations ranged from 4.60 to 12.80 mm, respectively (Figure 1D, Table 1). A normal frequency distribution of SL was observed in the developed F_2_ mapping populations grown in two experimental years (2020 and 2021) (Figure 2G,H). In addition, it was noticed that the obvious seed length of two parental lines was equally inherited in the resultant F_1_ as well as F_2_ segregating populations, demonstrating the polygenic dominance of the quantitative trait of SL.

#### 2.1.5. Seed Width (SWi, mm)

There were significant differences in seed width (mm) among the P_1_ (4.58 ± 0.16) and P_2_ (5.69 ± 0.25), and F_1_ (5.9 ± 0.08) (Figure 1B). The average SWi mean value of the 2020-F_2_ segregating population was 5.74 ± 0.84, and variations ranged from 3.80 to 10.10 mm. The SWi mean value of the 2021-F_2_ segregating population was 5.92 ± 0.80, and variations ranged from 3.90 to 9.10 mm, respectively (Figure 1D, Table 1). A continuous frequency distribution of SWi was observed in the developed F_2_ mapping populations grown in two experimental years (2020 and 2021) (Figure 2I,J), which showed the mutual genetic inheritance of parents and their F_1_ offspring. specifying the SWi as a quantitative trait with major-effects of polygenes.

#### 2.1.6. Seed Shape Index (SSi, SL/SWi)

There were significant differences in seed shape index among the P_1_ (2.08 ± 0.09), P_2_ (1.57 ± 0.08), and F_1_ (1.63 ± 0.03) (Figure 1B); however, the average SSi mean value of the 2020-F_2_ plants was 1.63 ± 0.12 and low to high variations were noticed in 1.38~1.99 mm range, and the SSi mean value of the 2021-F_2_ plants was 1.63 ± 0.18 and variations were noticed in 0.50~2.14 mm range, respectively (Figure 1D, Table 1). The SSi of the F_2_ mapping population presented a normal distribution in both experimental years (2020 and 2021) (Figure 2K,L), which indicated the quantitative genetics of seed shape index.

#### 2.1.7. Seed Weight (SW, g)

There were no substantial differences in the 1000-seed weight (g) of both parents “P_1_ (47.07 ± 1.61) and P_2_ (48.23 ± 0.86)” but F_1_ depicted the differentiated seed weight (62.90 ± 1.61). However, the average SW mean value of the 2020-F_2_ segregating population was 74.62 ± 34.53 and variations ranged from 27.33 to 209.33 g. The SW mean value of the 2021-F_2_ mapping population was 61.02 ± 18.49 and variations ranged from 20 to 117 g, respectively (Table 1). In addition, a normal distribution of SW frequency was observed in the respective F_2_ segregating population developed over two experimental years (2020 and 2021) (Figure 2M,N). It is supposed that 1000-seed weight was mainly controlled by quantitative genetics with polygenic effects index.

### 2.2. Analysis of Phenotypic Correlation

**Figure 3:**
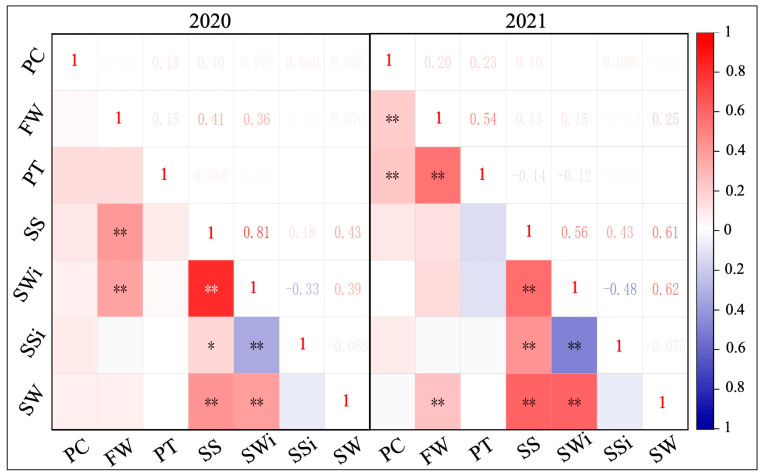
Pearson’s correlation analysis of fruit and seed phenotypes observed in two biparental (F_2_) mapping populations developed over two years (2020 and 2021). Correlation is significant at both statistical levels (* *p* < 0.05 and ** *p* < 0.01), respectively. The full names of abbreviations are as follows: fruit weight (FW), peel thickness (PT), seed length (SL), seed width (SWi), seed shape index (SSi), and weight of 1000 seeds (1000-SW).

The collected datasets of F_2_ phenotypes showed pretty stable results of correlation as shown in the visualized Pearson’s correlation matrix (Figure 3). In 2020, FW was highly correlated with SL and SWi, having coefficient values of 0.44 and 0.36, respectively. The correlation coefficient between FW and SW in 2021 was 0.25, demonstrating that seed traits had a direct impact on fruit weight. Further, FW and PT demonstrated a highly significant and positive correlation with coefficient values of 0.54, indicating that peel thickness also affected the weight per fruit. A significant and positive correlation of PC with FW and PT (0.20 and 0.23) was also observed. Regarding the seed traits, a significant correlation was noticed among SL, SWi, SSi, and SW. In 2020 and 2021, SL and SWi had extremely significant and positive correlations with correlation coefficients of 0.81 and 0.56, respectively, although SL and SW similarly showed extremely significant and positive correlations with coefficient values of 0.43 and 0.61, individually. SL and SSi also disclosed a very significant positive correlation with correlation coefficients of 0.18 and 0.43, respectively.

### 2.3. Construction of Genetic Linkage Map

**Figure 4:**
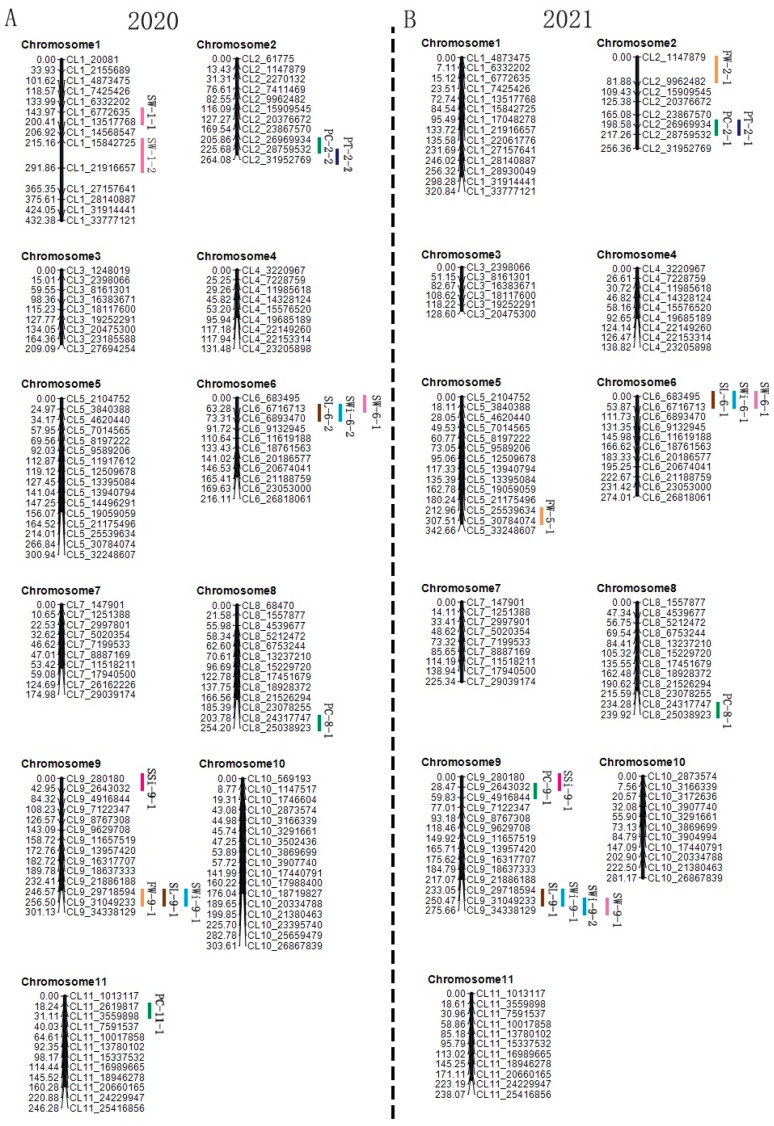
Genetic linkage mapping and QTL analysis of fruit and seed traits, based on CAPS marker genotyping. (**A**) QTLs identified in the 2020-F_2_ mapping population (**B**) QTLs identified in the 2021-F_2_ mapping population. The genetic distance in centimorgans (cM) is on the left side of each chromosome, the physical location is on the right side of each chromosome, and the vertical color bars represent the QTL sites of each trait.

Firstly, a high-quality genomic DNA was extracted (Supplementary Figure S1) and the average depth (20×) of parent genome re-sequencing revealed 80,121,269 and 80,005,427 clean reads with Q20 values and 98.50% and 98.55% of reference genomic coverage for each parent, respectively (Supplementary Table S2). A total of 3,609,977 SNPs were mined from the resequencing data which confirmed the screening of codominant CAPS markers among P_1_, P_2_, and F_1_ genotypes (Supplementary Figure S2). Two genetic linkage maps were successfully constructed using 136 codominant polymorphic CAPS markers genotyped within a 133-F_2_ population developed in 2020 and 119 polymorphic CAPS markers genotyped within a 250 F_2_ mapping population in 2021 (Supplementary Figure S3, Figure 4).

The genotyped markers were consistently positioned on the 11 chromosomes, as shown in the constructed linkage maps (Table 2, Figure 4). The physical positions of CAPS markers also indicated a good-fit Mendelian segregation ratio (1:2:1). In 2020, chromosome 10 had the maximum markers (n = 17), covering 20,081–33,777,121 bp in physical location, 303.61 cM in genetic distance, and 17.86 cM in average genetic distance between markers. In 2021, chromosome 6 had the less markers (n = 6), with a physical location of 683,495–26,818,061 bp, a genetic distance of 274.01 cM, and an average genetic distance between markers of 24.91 cM. The total length of the genetic linkage maps constructed in both years (2020 and 2021) was 2834.28 cM and 2721.45 cM, with average distances of 20.84 cM and 22.98 cM, respectively. The codominant flanking markers showed good marker-trait association between physical location and genetic distance, which effectively helped for QTLs and genes identification in the F_2_ mapping population.

### 2.4. Mapping of QTLs

QTLs were mapped using the fruit and seed-related phenotypic values of seven traits and the marker genotypic data of the F_2_ population developed over two years. In 2020, a total of 13 QTLs were dissected for the seven traits by genotyping 136 polymorphic markers within a 133-F_2_ population. In 2021, a total of 14 QTLs were spotted using the genotyping of 119 CAPS markers within 250 F_2_ populations. A total of 22 QTLs were identified; among them, five major-effect QTLs (PC-8-1, SL-9-1, SWi-9-1, SSi-9-1, and SW-6-1) were found to be colocalized over two years (2020 and 2021). However, four markers controlling the same trait were closely positioned next to each other (PC-2-1 and PC-2-2; PT-2-1 and PT-2-2; SL-6-1 and SL-6-2; and SWi-6-1 and SWi-6-2). The genetic position, LOD value, PVE%, additive effect, and dominant effect of each detected QTL at a given chromosome location are shown in Table 3 and Figure 4. A significant allele-specific contribution of parental lines and F_1_ offspring was also observed for inherited genetic effects among the detected QTL markers (Supplementary Figure S4).

#### 2.4.1. QTLs of PC

A total of five QTLs (two major-effect and three minor-effect) of PC (PC-2-1, PC-2-2, PC-8-1, PC-9-1, and PC-11-1) were located across four different chromosomes (Chr02, Chr08, Chr09, and Chr11). The major-effect QTL (PC-8-1) was situated between the two flanking markers (CL8_24317747 and CL8_25038923) with a physical distance of 721.17 kb. The LOD values were 23.34 and 22.28 and the PVE values were 28.29% and 38.98% over both experimental years (2020 and 2021), separately. Two other QTLs “PC-2-1 (2021) and PC-2-2 (2020)” were located among the adjacent markers (CL2_26969934 and CL2_28759532) positioned on chromosome 2. The LOD values were 7.24 and 9.18, and the PVE values were 8.50% and 10.00%, respectively. Two minor-effect QTLs (PC-9-1 and PC-11-1) were mapped across two years, with LOD values of 5.08 and 3.43 and PVE values of 8.97% and 4.01%, respectively. This indicated that the watermelon peel color gene was obviously controlled by a candidate gene, and the detection of QTLs across Chr02, Chr08, and Chr11 indicated that the peel color is unlinked and controlled by multiple genes. So, we hypothesized that the major-effect QTL (PC-8-1) was found to be a stable QTL regulating the peel color, while the genetic positions of minor-effect QTLs (PC-2-1 and PC-2-2, PC-9-1 and PC-11-1) were changed in different years due to natural environmental effects (years).

#### 2.4.2. QTLs of FW

A total of three QTLs of FW (FW-2-1, FW-5-1, and FW-9-1) were found on three different chromosomes (Chr02, Chr05, and Chr09) that had major and minor-effects during the two experimental years. In 2020, the major-effect QTL (FW-9-1) was found between markers CL9_29718594 and CL9_31049233 with a physical distance of 1.33 cM, a LOD value of 6.29, a PVE of 20.12%, and a positive additive effect, which showed the genetic effect of increased single fruit weight. The minor-effect QTLs (FW-2-1 and FW-5-1) were mapped in a single year (2021). These two QTLs were found on Chr02 and Chr05, with LOD values of 3.12 and 6.18 and PVE values of 6.92% and 9.94%, respectively. It was hypothesized that the positive additive effects of FW-9-1 and FW-2-1 are supposed to increase the single fruit weight, while the negative additive effect of FW-5-1 decreases the single fruit weight. The identified QTLs indicated that the single fruit weight trait of watermelon is also regulated by polygenes and supposed to be greatly influenced by environmental factors (years).

#### 2.4.3. QTLs of PT

A total of two QTLs of PT “PT-2-1 (minor-effect) and PT-2-2 (major-effect)” were identified on chromosome 2 (near the CL2_28759532 marker) over the two experimental years (2020 and 2021), separately. The minor-effect QTL (PT-2-1) was positioned between adjacent markers (CL2_26969934 and CL2_28759532), with a physical distance of 1.78 cM, a LOD value of 3.57, a PVE of 7.69%, and a positive additive value that indicated the genetic effects of greater peel thickness. The other major-effect QTL (PT-2-2) was located between the markers (CL2_28759532 and CL2_31952769), with a physical distance of 1.33 cM, a LOD value of 5.25, a PVE of 17.04%, and a negative additive value that indicated genetic effects of less peel thickness. The above results indicated that watermelon peel thickness traits were mainly affected by one pair of genes but were less affected by environmental factors (years).

#### 2.4.4. QTLs of SL

A total of three major-effect QTLs of seed length (SL-6-1, SL-6-2, and SL-9-1) were localized on two different chromosomes (Chr06 and Chr09) over both experimental years (2020 and 2021). The SL-9-1 was found to be a stable QTL between two nearby markers (CL9_29718594 and CL9_31049233) at a genetic position of 1.33 cM, with LOD values of 11.59 and 11.66 and PVE values of 18.78% and 18.93%, respectively. This QTL depicted the positive additive effect (0.09) for increased length of the seed in the year 2020 and the negative additive effect (−0.94) for reduced seed lengths in the developed mapping populations. The genetic locus of SL-6-1 (2021 year) and SL-6-2 (2020 year) were tightly localized to each other over both years and located between genetic markers (CL6_683495 and CL6_6716713, and CL6_6716713 and CL6_6893470) positioned on Chr06 with LOD values of 6.93 and 6.66, and PVE values of 11.27% and 15.16%, respectively. These results indicated that the watermelon seed length traits were mainly affected by two pairs of genes, that the two pairs of genes were not linked in the genome, and that environmental factors (years) had little influence.

#### 2.4.5. QTLs of SWi

A total of four QTLs of SWi (three major-effects (SWi-6-1, SWi-6-2, and SWi-9-1) and one minor-effect (SWi-9-2)) were mapped on two different chromosomes (Chr06 and Chr09). The major-effect QTL of SWi-9-1 was found co-localized in both experimental years (2020 and 2021), positioned between two markers (CL9_29718594 and CL9_31049233), with a physical distance of 1.33 cM, LOD values of 12.91 and 2.67, and PVE values of 34.81% and 3.77%, respectively. This QTL (SWi-9-1) showed a positive additive effect value (0.07) for the genetic effect of more seed width in 2020 and a negative additive effect value (−0.33) for reduced seed width in 2021. Another QTL (SWi-9-2) seemed to be linked to the major-effect QTL (SWi-9-1) and was located between two nearby markers (CL9_31049233 and CL9_34338129), with a LOD of 8.42 and a PVE of 11.27%. Therefore, it is suspected that there may be key genes affecting seed width near the CL9_31049233 marker. On Chr06, the major-effect locus (SWi-6-1, found in 2021) and the minor-effect locus (SWi-6-2, found in 2020) were spotted close to each other within the markers (CL6_683495 and CL6_6716713) and seemed near the CL6_6716713 marker. In 2020 and 2021, LOD values were 4.31 and 4.70, and PVE values were 9.67% and 10.68%, respectively. These results indicated that the watermelon seed length traits were mainly affected by two pairs of genes, which were less affected by environmental factors (years).

#### 2.4.6. QTLs of SSi

A major-effect QTL for SSi (SSi-9-1) was found on chromosome 9. It should be noted that SSi-9-1 was a stable QTL that was found in both experimental populations grown in 2020 and 2021. It was found between two nearby markers (CL9_280180 and CL9_2643032) at a physical distance of 2.36 cM with LOD values of 7.72 and 7.21 and PVE values of 23.14% and 12.06%, respectively. In 2020, the SSi-9-1 QTL had a negative additive effect (−0.09), which meant that the seed shape index was smaller; however, in 2020, this QTL was found to have a positive additive effect (0.12), which meant that the seed shape index was increased. These findings suggested that a pair of key genes controlled the watermelon seed shape index and that environmental factors (years) had a less significant impact.

#### 2.4.7. QTLs of SW

A total of four SW QTLs (SW-1-1, SW-1-2, SW-6-1, and SW-9-1) were spotted with major and minor-effects across three different chromosomes (Chr01, Chr06, and Chr09). One major-effect locus (SW-6-1) was found to be a stable QTL that was co-localized over two years of experimentation, with LOD values of 4.64 in 2020 and 4.21 in 2021 and PVE values of 5.33% in 2020 and 13.30% in 2021. This QTL was located between two genetic markers (CL6_683495 and CL6_6716713) with a physical distance of 6.03 cM. QTL (SW-9-1) was located between two flanking markers (CL9_31049233 and CL9_34338129), with a physical distance of 3.28 cM, a LOD value of 8.75, and a PVE of 15.08%. Two minor-effect QTLs (SW-1-1 and SW-1-2) were identified in a single year of 2020 and located between genetic markers (CL1_6772635 and CL1_13517768, CL1_15842725 and CL1_21916657) and positioned on Chr01, with LOD values of 3.35 and 3.68 and PVE values of 6.15% and 7.40%, respectively. These results showed that 1000-seed weight was controlled by major gene pairs and various minor genes. However, the multiple genes were not linked in the genome and were supposed to be less affected by environmental factors (years).

### 2.5. Analysis of Putative Genes Underlying Major QTLs

**Figure 5:**
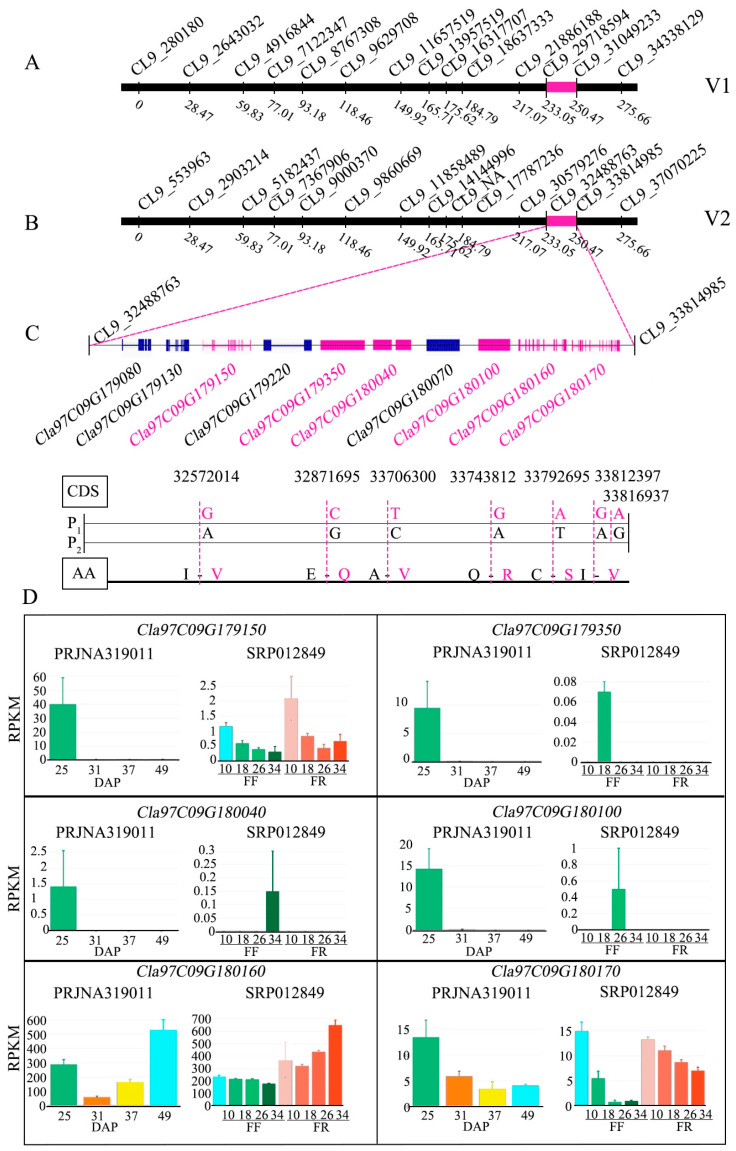
Prediction and analysis of candidate genes underlying QTLs of seed morphology of watermelon. (**A**) Genetic segments of seed size (length and width) QTLs mapped on a developed genetic linkage map in 2021, using the watermelon (97103) reference genome “v1”. (**B**) Watermelon reference genome “97103, v2”. (**C**) Candidate gene mutations in the CDS region led to the existence of non-synonymous amino acid mutations in genetic regions of both parental lines. (**D**) Candidate genes expression analysis in different tissues sampled at different time periods.

Mainly, we identified the major putative QTLs for fruit and seed traits, but we decided to check the putative genes underlying the pretty stable seed size-related QTLs (SL-9-1 and SWi-9-1), which were co-localized between two nearby markers (CL9_29718594 and CL9_31049233) on Chr09, over two experimental years (Figure 4 and Figure 5A,B). The multiple sequences of comparative parent lines and the reference genome (97103, v2) assembly were aligned, and candidate mutations in the target interval of CDS regions and the amino acid sequence of ten annotated genes underlying the QTL region were identified (Supplementary Table S3).

According to the visual seed characteristics (Figure 1B), P_1_ is slim-shaped with a low seed weight, P_2_ is oval-shaped with a higher seed weight (Table 1), and the seed characteristics of 97103 watermelon are the same as those of P_2_ (Supplementary Figure S5). Interestingly, the multiple sequence alignment revealed the presence of candidate base mutations in the gene CDS site of P_2_ sequences and non-synonymous mutations found in six genes: *Cla97C09G179150* (A-G), *Cla97C09G179350* (G-C), *Cla97C09G180040* (C-T), *Cla97C09G180100* (A-G), *Cla97C09G180160* (T-A), and *Cla97C09G180170* (A-G to G-A), but a total of four genes with non-synonymous mutations were found (Supplementary Table S3). The gene structural variation were analyzed and protein sequence alignment showed mutations in the amino acid sequences of the following genes: *Cla97C09G179150* (I-V), *Cla97C09G179350* (E-Q), *Cla97C09G180040* (A-V), *Cla97C09G180100* (O-R), *Cla97C09G180160* (C-S), and *Cla97C09G180170* (I-V) (Supplementary Figure S6, Figure 5C).

Further, the gene expression levels were checked using the publicly available transcriptomic database. We found that four genes (*Cla97C09G179150*, *Cla97C09G179350*, *Cla97C09G180040*, and *Cla97C09G180100*) were mainly downregulated in watermelon seeds, especially at 25 days after pollination “DAP” (Figure 5D). These four genes were identified as important candidate genes for seed development, indicating that the seed morphology had been formed in the early stages. However, two other genes (*Cla97C09G180160* and *Cla97C09G180170*) were obviously expressed in seed, peel, and flesh tissues, which showed that their expression patterns were inconsistent with expectations, so they were excluded.

## 3. Discussion

In recent years, whole genome resequencing has emerged as the most practical and widely used omics-based technology for studying genetic differences between materials of the same species [30]. It effectively reduces the time and effort of plant breeders and scientists in developing codominant molecular markers, pinpointing the major-effect locus conferring multivariate phenotypes [31]. In 2020, the genome of watermelon variety was further improved (97103, v2), with more than 69.5 million effective reads comprising 362.7 Mb of genome coverage for the 11 chromosomes [32], intersecting with the earlier published reference genome (v1), but the number of genes decreased to only about 22,596 genes, the sequencing was deeper, wider, and more accurate. Therefore, in this study, we selected the latest reference genome (v2) data for comparing the coverage of parental genome resequencing, which was not only used for SNP mining but also for candidate gene prediction. Two different cultivars revealed an average depth of parental sequencing (20× depth), and 80,121,269 and 80,005,427 clean reads were obtained with Q20 values of 98.50% and 98.55% coverage for each parent (Supplementary Table S2). A total of 3,609,977 high-quality SNP loci were obtained, which ensured the development of codominant CAPS markers. The molecular genotyping in two respective segregating populations depicted the accuracy of the genotyping and two linkage maps identified a total of 22 QTLs for watermelon fruit and seed traits (Figure 4).

Fruit weight (FW) is known as an important commercial trait, and different botanical cultivars of watermelon range in weight from g to kg, depending upon size [12]. In previous studies, multiple QTLs have been dissected across Chr03, Chr08, and Chr09 [1,7,8,11,33,34,35]; However, a recent study exposed the novel QTLs situated over Chr06 and Chr07, controlling the fruit weight of watermelon [12]. In this study, two segregated F_2_ mapping populations were derived over the course of two years and found three major-effect QTLs (FW-2-1, FW-5-1, and FW-9-1) on three different Chr02, Chr05, and Chr09, and the PVE% was varied, dependent on how much the fruit weight changed (Table 3, Figure 4). The QTL results of Chr09 are consistent with the previous study results, but the Chr02 and Chr05 QTLs contradict each other, suggesting that the genetic deviations in the parental lines and their developed segregated populations are the main reason for the regulation of differential genetic regions. Further, it was also noticed that FW is a multifaceted quantitative trait that is synchronized by polygenes (Figure 2). For genetic regulation, it has been stated that the LC (YABBY, FAS, and WOX family members) is the main reason for triggering the gradual changes in fruit weight [34]. The FW2.2 was similarly identified as a major-effect QTL regulating fruit weight by encoding the protein of cell number regulators “CNR” [36]. So, we hypothesized that our spotted QTL sites might have potential genes, but solid justification is still required through map-based cloning studies.

Fruit peel color (PC) is an appealing visual trait for identifying the commercial maturity stages of watermelon. The edible watermelon peels are varied in color (solid-green, light-green, and green-yellow), and numerous studies have reported that dominant and recessive genes (*G* > *gs* > *g*) have strong allelic relationships for peel color [13,35,37,38]. The dark green peel color was known as dominant over the light green peel with stripes (*gs*) [39] and incompletely dominant over the yellowish-green or gray peel [40,41,42]. In this study, the good-fit Mendelian segregation inheritance of peel color was observed in two F_2_ mapping populations, consistent with the genetic patterns of the parental genotypes (Figure 2, Supplementary Table S1). Up to now, only a few genetic studies have significantly shown that Chr08 is mainly controlling the genetic regulation of watermelon peel color [39,43]. In our study, whole genome CAPS markers exposed a total of 5 potential genomic regions (QTLs) affecting the peel color of watermelon at dissimilar genetic locations across Chr02, Chr08, and Chr09 (Table 3, Figure 4). The PC-related QTLs (PC-2-1, PC-2-2, and PC-8-1) were mapped with major and minor effects, LOD values, and PVE across two consecutive years. These detected QTLs indicated that the peel color is regulated by unlinked genomic architecture and polygenic phenomena. Our study identified the PC-related consistent and stable QTL (PC-8-1) positioned on Chr-08 that exhibits strong validation of Park et al., [39] and Li et al., [43]. However, the other consistent genetic segment of Chr08 was discovered with the presence of ~262 kb cutting site in the watermelon genome (97102, v1) [43], which exposed the potential gene (*ClCGMenG*), encoding 2-phytyl-1,4-beta-naphthoquinone methyltransferase. Regarding the PC-related QTLs positioned on Chr02, a novel major-effect QTL regulating rind color (FRC-2.1) situated at the lower end segment of Chr02 has been reported [12], which is also consistent with our mapped QTLs (PC-2-1 and PC-2-2) located on Chr02. The differences in genetic position depicting the novel QTLs and the existence of additional genes controlling peel color in watermelon. A recent study also classified that the fruit peel color is governed by a genetic locus positioned between two closest SNP markers at the bottom end segment of Chr08 [8]. Hence, we think that our identified novel positions of QTLs controlling the peel color trait need to be delimited for additional gene functional validations.

Fruit peel thickness (PT) is generally linked with storage life, susceptibility or resistance, and splitting or cracking of fruit [10,33,44]. To date, few molecular studies have shown the molecular regulation of watermelon peel thickness. Genetic segregation analysis showed that peel thickness is primarily regulated through a candidate region located at the Chr09 segment [33]. A recent QTL mapping study described that watermelon peel thickness is mainly controlled by a potential genomic region (FRT-4.1) positioned on Chr04 [12]. The previous molecular study showed that one major-effect QTL (RTH-2.1) of peel thickness was mapped on Chr02 [11], which explained 14.74% of the major PVE for the less fruit peel thickness and dominance of parental alleles observed. However, in this study, we mapped two QTLs (PT-2-1 with a minor-effect and PT-2-2 with a major-effect) over two years (Figure 4), which explained a total of 24.73% PVE for thick and thin peel thickness (Table 3). Apparently, our spotted QTLs contradict other studies, therefore exposing the new genomic regions controlling peel thickness. Further, the phenotypic analysis indicated that the peel thickness trait is a quantitative trait that is mainly controlled by a major-effect gene; however, a polygenic phenomenon is involved in regulating the thickness variation levels (Figure 2). Regarding the genetic and physiological basis, differential watermelon peel thickness was reported to be connected with consistent peel hardness, cracking resistance or susceptibility [44], and fruit weight [10]. It was shown that low to high peel thickness levels occur due to the different cell sizes and shapes, as well as the accumulation of endogenous lignin in the cell walls, and class III peroxidase genes have been validated for regulating internal cell structure and lignin [11]. Similarly, eggplant peel with more firmness depicted an appropriate hindrance to fruit cracking due to the thick peel structure, which also affected persistent storage life and consumption period [45]. We think that our spotted QTLs for PT might exhibit potential genes and significant expression profiling, but in-depth and valid justification is still required through map-based cloning and gene functional studies.

Seed is known as a main part of the growth and development of plants but the genetics of seed traits have been less studied in vegetable crops [12]. Watermelon seed size is strongly assumed to be governed by a single dominant gene [46]; however, small to large seed sizes have been reported [12,20]. Until now, multiple QTLs of watermelon seed-associated traits have been spotted with significant phenotypic variances across the genome-wide chromosomes (1, 2, 3, 4, 5, 6, 7, 8, 9, 10) using various segregated populations developed from crossing of wild type and improved cultivar lines [1,8,12,16,20,33,47,48,49,50,51,52,53,54,55]. In our study, four QTLs of seed weight (SW-1-1, SW-1-2, SW-6-1, and SW-9-1), three QTLs of seed length (SL-6-1, SL-6-2, and SW-9-1), four QTLs of seed width (SWi-6-1, SWi-6-2, SWi-9-1, and SWi-9-2), and one stable QTL of seed shape index (SSi-9-1) were spotted on distinct chromosomes (Table 3, Figure 4). The QTL results of Chr04, Chr06, Chr07, and Chr11 are consistent with the above-mentioned molecular mapping studies, except for the differentially identified genetic positions. Further, the detected QTLs (SL-9-1, SWi-9-1, and SW-6-1) justified the stable genomic regions for controlling the seed length, width, and weight. The QTL results of seed phenotypes seemed to be the same and supported the quantitative distribution of seed trait segregation (Figure 2), which shows how parental lines and offspring are shifted from seed to seed in the F_2_ mapping populations.

As far as we know, few molecular studies have been conducted regarding the in-depth genetic evaluation of seed-associated traits in watermelon. The genetic inheritance of size and shape of watermelon seeds was primarily evaluated, and it was found that small-to-large-sized seeds depict a major dominance over medium-sized seeds [20]. The large and small seed sizes seemed to be genetically controlled by candidate genes (*s* and *l*), and the *Ti* gene for tiny seed and the *ts* gene for tomato seed were identified as having major-effects on small-sized seed regulation [20]. One major-effect locus (*qSS6*) was mapped on Chr06, regulating the genetic region responsible for controlling watermelon seed size and containing three candidate genes (*Cla009291*, *Cla009301*, and *Cla009310*), as reported by Li et al., [16]. *Cla009291* gene was highly homologous to the gene *srs*, which controls rice seed size and contains a kinesin motor domain. In addition, *Cla009291* gene was shown to encode the MDR-protein (mdtK), which differentially showed higher expression during the growth stages of large and small seeds of watermelon varieties. The *Cla009310* gene was depicted as a candidate gene, encoding an unknown protein for regulating the seed size and shape between comparative watermelon lines, and also identified as a homolog of *SRS3* (SMALL AND ROUND SEED) for a kinesin-13 like protein, known as the seed size regulator in rice [56]. Another *Cla007520* gene was found to be expressed higher during the initial stage of seed development. This gene was found to be a member of the cell-penetrating peptide (CPP)-conjugated protein family and to have amino acids that are similar to those of AtTSO1, which is responsible for cell division during the ovule development stage [57]. In our study, the spotted QTLs of seed traits (SL-6-1 and SL-6-2, and SWi-6-1 and SWi-6-2) were found at the same genetic location, but SL-9-1 and SWi-9-1 were exhibited as dominant stable regions controlling the seed size trait and positioned on Chr09 in over two experimental years (Figure 4). Thus, we supposed there might be the existence of major-effect genes that could exhibit substantial expression for seed size formation, and we focused on analyzing the putative genes underlying seed-related pretty stable QTLs (SL-9-1 and SWi-9-1) (Figure 5A,B). A total of four new candidate genes (*Cla97C09G179150*, *Cla97C09G179350*, *Cla97C09G180040,* and *Cla97C09G180100*) showed significant involvement in the regulation of seed shape difference and seed morphology of watermelon (Figure 5). Among them, *Cla97C09G179150* function annotation is long chain acyl-CoA synthetase 2, *Cla97C09G179350* function annotation is ethylene-responsive transcription factor 1-like, *Cla97C09G180040* and *Cla97C09G180100* function annotation are metalloendoproteinase 2-MMP-like (Supplementary Table S3, Figure S6). In addition, our resequencing results of the two watermelon materials revealed that there were non-synonymous type mutations in the CDS region and amino acid sequences of these four genes (Figure 5C), which may cause changes in gene function and further affect seed development. A transcriptomic study revealed the expression levels of these 4 genes, which had certain tissue specificity (seed > pulp > peel) and gradually stopped with the formation of seed morphology, and also indicated that these gene were mainly involved in seed development (Figure 5D).

It has been stated in former studies that LACS-2 (EC 6.2.1.3) triggers the ATP-dependent activation of free fatty acids to form acyl-coenzymes, which are major acyl-donors in various lipid metabolic pathways [57]. The increased transcripts of the *LACS2* gene promote the expression level of glycolysis-, FA-, and lipid synthesis-related genes during seed development [58]. Similarly, it was reported that long-chain fatty acids in seeds and stored fat cleave acetyl-CoA molecules several times. Acetyl-CoA can then enter the citric acid cycle and eventually be oxidized for energy production [58]. In this study, we also identified that the *Cla97C09G179150* gene harbor non-synonymous SNP (A-G) and amino acid sequence mutation (I-V), encoding Long chain acyl-CoA synthetase 2 (Supplementary Table S3 and Figure S6). It is therefore strong possibility in that the *Cla97C09G179150* gene might have involvement in seed development of watermelon. In addition, ERF was also found to have a crucial effect on controlling seed size development in soybean [59,60]. The genetic regulation impact of *AP2* on embryo, endosperm, and seed coat development mainly determined the seed size of *A. thaliana*; *AP2* controlled the seed quality of the maternal line; and the *AP2* mutant produced larger-sized seeds than those of the wild-type line [61]. Moreover, it was found that the *APETALA2* transcription factor “SNB (SUPERNUMERARY BRACT)” regulated the seed size of *Oriza sativa* [62]. Its increased glume longitudinal cell elongation causes the *ssh1* mutant to produce a higher density and grain weight of seed compared to the wild type. In our research findings, we noticed the significant gene expression profiling of *Cla97C09G179350* at initial developmental stage of 25 DAP and assumed that this gene might have important involvement in the regulation of seed morphology of watermelon (*Citrullus lanatus* L.); however, at present, the in-depth regulatory function of both *Cla97C09G180040* and *Cla97C09G180100* genes has not been reported in relation to seed formation which needs to be validated.

## 4. Materials and Methods

### 4.1. Experiment Materials and Mapping Populations

Two different inbred lines of watermelon (*Citrullus lanatus* L.) “P_1_ (1061, female parent, fruit with dark green peel color, thick peel, and small to slim seed size) and P_2_ (812, fruit with light green peel color, thin peel, and large to oval seed size)” were used as plant materials. The pictorial view of fruit and seed morphology differences can be seen in Figure 1. The inbred parental lines were kindly provided by the Zhengzhou Fruit Research Institute (ZFRI) of the Chinese Academy of Agricultural Sciences (CAAS), Zhengzhou, China. Then, these lines were crossed to obtain F_1_ hybrid and F_2_ mapping populations, and collected seeds were further stored in the germplasm bank of Laboratory of the Molecular Genetics and Breeding, Horticulture College, Northeast Agricultural University (NEAU), Harbin, China.

A two-year biparental (F_2_) mapping population was cultivated in the plastic greenhouse (45°07′ N, 125°430′ E) of the Xiangyang Agricultural Station of NEAU. In 2020 year, a total of 20 plants of both parental lines, F_1_ hybrids, and 133 plants of the F_2_ mapping population were planted. In 2021 year, a total of 20 plants of both parental lines, F_1_ hybrids, and 250 plants of the segregated F_2_ population were planted for the repeated experiment, respectively. To ensure the proper growth of plants, a mixture of 60% loamy soil, 30% compost, and 10% potting mixture (peat moss, perlite, and vermiculite) was used in the raised beds of the greenhouse. The plant cultivation was done in a randomized complete block design (RCBD) by maintaining the P×P (70 cm) and R×R (60 cm) distances on greenhouse ridges for well growth under natural light conditions. The day temperature was 25–30 °C, and at night it was 18–25 °C with 30–85% relative humidity, except for the occasional harsh weather condition during the whole experiment. The two-vine pruning and regular cultivation practices (irrigation, weeding, and hoeing) were applied for better growth and development of the crop plants.

### 4.2. Phenotyping of Fruit and Seed Traits

In both experimental years, female flowers at the second and third nodes of the 133 F_2_ and 250 F_2_ plant populations were checked on a daily basis and manually pollinated. All the mature fruits were collected at 35–45 days after pollination (DAP), and fruit and seed traits “peel color (PC), peel thickness (PT), fruit weight (FW), seed length (SL), seed width (SWi), seed shape index (SSi), and 1000-seed weight (SW)” were investigated, respectively. In brief, PC was checked by visually observing the rind color “dark green (scored as 1), light green (scored as 3), and intermediate (scored as 2)”. FW was determined in kilograms (kg) units by using an electronic weight balance machine. Then, fruits were cut in a vertical shape, and PT was calibrated in millimeters (mm) by checking the internal gap of the exocarp and mesocarp layer of peel. Seeds were removed from the edible flesh portion of the fruit, dried in the shaded area, and phenotyping of seed morphology was done. The weight of 1000 seeds (SW) was measured in grams (g) using the mini electronic weighing machine. The maximum length of the seed (SL) was checked at the longest axis of the seed, and the maximum width of the seed (SWi) was checked at the widest axis of the seed by using the electronic calibrated ruler. The seed shape index (SSi) was measured in ratio by dividing the seed length over the seed width (SL/SWi).

### 4.3. Whole Genome Resequencing (WGRS)

The disease-free young leaf samples were collected from the F_2_ plant populations, F_1_ hybrids, and parent materials, put into 2 mL tubes, and frozen at an ultra-low temperature. Total DNA was isolated from the respective samples using the slightly modified CTAB method and quantified by 1% agarose gel electrophoresis. Whole genome resequencing was performed using the Illumina HiSeq 2500 system at Beijing Genomic Institute (BGI), Beijing, China. The raw DNA-seq data (Sequence Read Archive (SRA), Accession: PRJNA915415, containing 2 biosamples) was submitted to the online NCBI website database. The bioinformatics analysis of raw DNA-seq reads was performed, SNP-CAPS markers were exported, and the co-dominance was identified based on the homozygous parents (P_1_ and P_2_) and heterozygous (F_1_), respectively.

In brief, the final WGRS reads were used to filter the paired end adapter sequences, and data processing steps were performed for quality adjustment. The filter parameters of SOAPnuke software “version 1.12” (released by BGI) were used for obtaining valid, clean end reads [63], as shown in Supplementary Table S2. The clean end bases sequenced from the opposite ends of both parental lines were compared with the improved genome of watermelon (97103, v2) by using the BWA software “version: 0.7.17” (http://www.ricugi.org/, accessed on 15 January 2020)) [64]. Then, the BAM files were employed for filter alignment of the resequencing bases, and the tagged identical bases were removed using the Picard tool (v1.106). The final BAM sequences were arranged, and the SAMtools analysis software (version 0.1.19) was used for the mapping of suitable sequence reads and SNP sites [65].

### 4.4. Development of CAPS Markers

According to the mapping statistics of parental genomic re-sequencing data, a total of 13,119 CAPS loci pairs were found to have proper cutting sites for 11 restriction enzymes, such as *EcoR* I, *Sca* I, *BsaH* I, *Hind* III, *Hind* II, *Pst* I, *Dal* I, *Msp* I, and *Bcl* I. These restriction enzymes uncovered the main polymorphic sites across all chromosomes (n = 11). In total, 15-20 suitable physical sequence sites of SNPs “before and after 500 base pairs (bp)” were selected and transformed into CAPS markers using the SNP2CAPS analysis tool [66]. Then, PCR-based novel CAPS markers were designed (Supplementary Table S4) based on the corresponding reference genomes of watermelon (v1.0 and v2.0) using Primer Premier (v6.0), as earlier reported [12]. All the transformed CAPS primer sequences were oligo-synthesized by Sangon Biotech Company and then labelled for the ease of experimentation, e.g., chromosome number and physical location, respectively. The optimized PCR reaction system and enzyme digestion reaction were completed using the formerly described genotyping protocol [12]. The PCR-yielded products were cut with a suitable restriction enzyme, and the co-dominant bands with different base pairs were cleaved on a 1% agarose gel using the imaging systems. Finally, a total of 136 and 119 pairs of CAPS markers were verified between the two different parents and F_1_ hybrids, over two experimental years, respectively.

### 4.5. Genetic Mapping and QTL Analysis

The coded dataset of allelic bands of total 136 CAPS markers (genotyped within 133 F_2_ mapping individuals developed in 2020 year) and a total of 119 CAPS markers (genotyped within 250 F_2_ mapping individuals developed in 2021 year) was used for the construction of two respective linkage maps and the identification of major and minor-effect QTLs using the IciMapping software (version 4.0) [67]. The genotyped markers were grouped across the whole genome of chromosomes by default setting, i.e., by a LOD threshold of 3.00, and ordered by the nnTwoOpt algorithm. The Kosambi mapping method was used to estimate the genetic intervals and positions of markers in centimorgans (cM) across each chromosome. A good-fit Mendelian segregation ratio of markers was noticed at a *p*-value (0.05), and linkage mapping was performed for QTL detection based on the default LOD value (2.50). The mapped QTLs were given short names that included the abbreviated name of the trait, chromosome number, and QTL number, respectively.

### 4.6. Predictions of Putative Candidate Genes

The Integrative Genomics Viewer (IGV, v2.12.2) software was used to search the SNP mutations in protein coding sequence (CDS) regions of candidate genes intervals, and the DNAMAN (v9.0) software was used to translate the gene sequence into amino acid sequences, which were then sequenced and aligned for multiple sequence analysis (Supplementary Table S3, Supplementary Figure S6). The annotation of the candidate genetic locus was performed using the publicly available dataset of Cucurbitaceae crop genome (http://www.cucurbitgenomics.org/ accessed on 15 January 2020). Two relevant transcriptome datasets (SRP012849 & PRJNA319011) were mainly selected, and candidate gene expression analysis was performed in different tissues of the seeds, flesh, and peel of watermelon.

### 4.7. Statistical Data Analysis

The numerical phenotypic values were initially recorded in Microsoft Excel Worksheet (v2019) and descriptive statistics (means, standard deviation, range, and frequency distribution) was calculated by using the statistical software “IBM-SPSS (v23.0)”. The Pearson’s correlation analysis was done and visualized with computer software “OriginPro (2021)”.

## 5. Conclusions

In summary, we can say that whole genome resequencing is an effective omics approach for developing cost-effective genetic markers and dissecting the candidate genomic regions controlling various traits in crop plants. Our two-year experimental findings illustrated stable and major-effect QTLs of fruit and seed associated traits as well as major genes (*Cla97C09G179150*, *Cla97C09G179350*, *Cla97C09G180040*, and *Cla97C09G180100*) regulating the seed size of watermelon. These robust results mainly provide a strong genetic basis for additional map-based cloning and advanced marker-assisted breeding strategies aimed at enhancing the quality traits of watermelon.

## Figures and Tables

**Table 1:** Statistical analysis of different phenotypes of watermelon parental lines (P_1_, P_2_), F_1_, and F_2_ mapping populations.

Phenotypes	P_1_ (1061)	P_2_ (812)	F_1_ (Hybrid)	F_2_ Population in 2020			F_2_ Population in 2021		
	Mean ± SD	Mean ± SD	Mean ± SD	Mean ± SD	Low	High	Mean ± SD	Low	High
FW (kg)	3.45 ± 0.23 ^a^	2.65 ± 0.06 ^b^	3.74 ± 0.24 ^a^	2.79 ± 0.99	0.90	7.18	1.85 ± 0.99	0.12	5.26
PT (mm)	11.44 ± 0.02 ^a^	5.89 ± 0.11 ^c^	10.64 ± 0.03 ^b^	11.21 ± 3.42	4.73	21.13	8.33 ± 3.53	2.26	18.35
SL (mm)	9.52 ± 0.07 ^a^	8.9 ± 0.08 ^b^	9.62 ± 0.14 ^a^	9.32 ± 1.20	6.40	12.30	9.61 ± 1.30	4.60	12.8
SWi (mm)	4.58 ± 0.16 ^b^	5.69 ± 0.25 ^a^	5.9 ± 0.08 ^a^	5.74 ± 0.84	3.80	10.10	5.92 ± 0.80	3.90	9.10
SSi (SL/SWi)	2.08 ± 0.09 ^a^	1.57 ± 0.08 ^b^	1.63 ± 0.03 ^b^	1.63 ± 0.12	1.38	1.99	1.63 ± 0.18	0.50	2.14
1000-SW (g)	47.07 ± 1.61 ^b^	48.23 ± 0.86 ^b^	62.90 ± 1.61 ^a^	74.62 ± 34.53	27.33	209.33	61.02 ± 18.49	20	117

Note: Statistical values represent the mean ± standard deviations of collected datasets. Different letters are indicating the significance among mean data values of each column, and there was no significant difference in the mean with the same letter (*p* ≤ 0.05, Duncan’s test). The full names of abbreviations are as follows: fruit weight (FW), peel thickness (PT), seed length (SL), seed width (SWi), seed shape index (SSi), and weight of 1000 seeds (1000-SW).

**Table 2:** Cleaved amplified polymorphism sequence (CAPS) markers distribution across two constructed genetic linkage maps of watermelon.

Chromosomes	Linkage Map of F_2_ Mapping Population in 2020			Linkage Map of F_2_ Mapping Population in 2021		
	CAPS Markers	Genetic Length (cM)	Average Distance (cM)	CAPS Markers	Genetic Length (cM)	Average Distance (cM)
Cla97Chr01	14	432.38	30.88	14	320.84	22.92
Cla97Chr02	11	264.08	24.01	8	256.36	32.05
Cla97Chr03	9	209.09	23.23	6	128.60	21.43
Cla97Chr04	9	131.48	14.61	9	138.82	15.42
Cla97Chr05	16	300.94	18.81	14	342.66	24.48
Cla97Chr06	11	216.11	19.65	11	274.01	24.91
Cla97Chr07	10	174.98	17.50	9	225.34	25.04
Cla97Chr08	13	254.20	19.55	12	239.92	19.99
Cla97Chr09	14	301.13	21.51	14	275.66	19.69
Cla97Ch10	17	303.61	17.86	11	281.17	25.56
Cla97Chr11	12	246.28	20.52	11	238.07	21.64
Total	136	2834.28	20.84	119	2721.45	22.87

**Table 3:** Genetic effects of fruit and seed related traits in F_2_ mapping populations developed over two experimental years. The full names of abbreviations are as follows: logarithm of odds (LOD), percentage variance explained (PVE), additive (Add), dominance (Dom), and confidence interval (CI).

QTL	Adjacent Markers		LOD	PVE (%)	Add Effect	Dom Effect	Left CI	Right CI	Year
PC-2-1	CL2_26969934	CL2_28759532	9.18	10.00	0.33	0.23	209.50	217.50	(2021)
PC-2-2	CL2_28759532	CL2_31952769	7.24	8.50	−0.47	0.26	221.50	233.50	(2020)
PC-8-1	CL8_24317747	CL8_25038923	22.28	28.29	0.57	0.48	238.50	239.00	(2021)
	CL8_24317747	CL8_25038923	23.34	38.98	−0.83	0.63	238.50	249.50	(2020)
PC-9-1	CL9_2643032	CL9_4916844	5.08	8.97	0.32	0.27	30.50	49.50	(2021)
PC-11-1	CL11_2619817	CL11_3559898	3.43	4.01	−1.39	−4.24	20.50	36.50	(2020)
FW-2-1	CL2_1147879	CL2_9962482	3.12	6.92	0.60	−0.84	30.50	62.50	(2021)
FW-5-1	CL5_25539634	CL5_30784074	6.18	9.94	−0.97	−0.46	228.50	258.50	(2021)
FW-9-1	CL9_29718594	CL9_31049233	6.29	20.12	0.43	0.75	251.50	264.50	(2020)
PT-2-1	CL2_26969934	CL2_28759532	3.57	7.69	1.56	−0.21	200.50	230.50	(2021)
PT-2-2	CL2_28759532	CL2_31952769	5.25	17.04	−0.22	0.08	217.50	238.50	(2020)
SL-6-1	CL6_683495	CL6_6716713	6.66	15.16	0.85	−0.56	0.00	20.50	(2021)
SL-6-2	CL6_6716713	CL6_6893470	6.93	11.27	−0.08	−0.02	63.50	72.50	(2020)
SL-9-1	CL9_29718594	CL9_31049233	11.59	18.78	0.09	0.10	250.50	255.50	(2020)
	CL9_29718594	CL9_31049233	11.66	18.93	−0.94	0.86	242.50	250.50	(2021)
SWi-6-1	CL6_683495	CL6_6716713	4.70	10.68	0.62	−0.33	0.00	23.50	(2021)
SWi-6-2	CL6_6716713	CL6_6893470	4.31	9.67	−0.04	−0.02	65.50	82.50	(2020)
SWi-9-1	CL9_29718594	CL9_31049233	12.91	34.81	0.07	0.06	250.50	258.50	(2020)
	CL9_29718594	CL9_31049233	2.67	3.77	−0.33	0.39	236.50	249.50	(2021)
SWi-9-2	CL9_31049233	CL9_34338129	8.42	11.27	−0.62	0.50	255.50	271.50	(2021)
SSi-9-1	CL9_280180	CL9_2643032	7.72	23.14	−0.09	0.07	0.00	15.50	(2020)
	CL9_280180	CL9_2643032	7.21	12.06	0.12	0.07	10.50	26.50	(2021)
SW-1-1	CL1_6772635	CL1_13517768	3.35	6.15	0.76	−1.38	159.50	179.50	(2020)
SW-1-2	CL1_15842725	CL1_21916657	3.68	7.40	−0.97	−1.34	240.50	256.50	(2020)
SW-6-1	CL6_683495	CL6_6716713	4.64	5.33	−1.13	−1.26	31.50	55.50	(2020)
	CL6_683495	CL6_6716713	4.21	13.30	0.12	−0.08	0.00	26.50	(2021)
SW-9-1	CL9_31049233	CL9_34338129	8.75	15.08	−0.13	0.10	251.50	269.50	(2021)

## Data Availability

Available upon request from the corresponding author(s).

## Supplementary Materials

The following supporting information can be downloaded at: https://www.mdpi.com/article/10.3390/ijms242115755/s1.
