# Supplementary material for: Molecular Mapping of Putative Genomic Regions Controlling Fruit and Seed Morphology of Watermelon
Source: Int J Mol Sci. 2023 Oct 30;24(21):15755. doi: 10.3390/ijms242115755 (PMC10650541; doi:10.3390/ijms242115755)
Supplement: Supplementary file 1 [file ijms-24-15755-s001.zip › Supplementary Figure S6.pdf]

Supplementary Figure S6 | Comparative analysis of gene and protein sequences controlling seed traits.

```
Cla97C09G179080_1061_    ATGACTGATTTTTCAGCTTTCCGAACGGGCGGTTTCAAAAGGAAAATGCCGCGCCCTCATTTTCACAAGCTTGTTCTCACCTCAACTATCCAAGCTAGGA    100
Cla97C09G179080_812_     ATGACTGATTTTTCAGCTTTCCGAACGGGCGGTTTCAAAAGGAAAATGCCGCGCCCTCATTTTCACAAGCTTGTTCTCACCTCAACTATCCAAGCTAGGA    100
Consensus                atgactgatttttcagctttccgaacgggcggtttcaaaaggaaaatgccgcgccctcattttcacaagcttgttctcacctcaactatccaagctagga

Cla97C09G179080_1061_    AATTGAGAATCCCAGAAACTTTTGTCAGGATGATCAGGGATGAGCTTTCAGCAGTTGCAACCCTTACTGTTCCTGATGGCCATGTTTGGCGTGTGGGTTT    200
Cla97C09G179080_812_     AATTGAGAATCCCAGAAACTTTTGTCAGGATGATCAGGGATGAGCTTTCAGCAGTTGCAACCCTTACTGTTCCTGATGGCCATGTTTGGCGTGTGGGTTT    200
Consensus                aattgagaatcccagaaacttttgtcaggatgatcagggatgagctttcagcagttgcaacccttactgttcctgatggccatgtttggcgtgtgggttt

Cla97C09G179080_1061_    GAGAAAAGCCGATAACAAATTTTGGTTTGAAGATGGATGGCAAGGATTTCTTGAGCATTACTCCATACGGGTTGGGTATTTATTAGTATTCAGATATGAG    300
Cla97C09G179080_812_     GAGAAAAGCCGATAACAAATTTTGGTTTGAAGATGGATGGCAAGGATTTCTTGAGCATTACTCCATACGGGTTGGGTATTTATTAGTATTCAGATATGAG    300
Consensus                gagaaaagccgataacaaattttggtttgaagatggatggcaaggatttcttgagcattactccatacgggttgggtatttattagtattcagatatgag

Cla97C09G179080_1061_    GGAAACTCAGCATTCTGTGTTTTTATCTTTAATCTTAATGCTTCTGAAATAAACTACCAATCAGCTGCTCTCAGTAGTAATCAAAGGAATAATTACAGCA    400
Cla97C09G179080_812_     GGAAACTCAGCATTCTGTGTTTTTATCTTTAATCTTAATGCTTCTGAAATAAACTACCAATCAGCTGCTCTCAGTAGTAATCAAAGGAATAATTACAGCA    400
Consensus                ggaaactcagcattctgtgtttttatctttaatcttaatgcttctgaaataaactaccaatcagctgctctcagtagtaatcaaaggaataattacagca

Cla97C09G179080_1061_    TTCAAAACCGAATCTTTGAAGAAATGGAGGATTATGACATTCCTGAAGCCATTCCCTCCAATCAGTCCATGAATTCCGGTTCTTTGCGAAATAAGCTATT    500
Cla97C09G179080_812_     TTCAAAACCGAATCTTTGAAGAAATGGAGGATTATGACATTCCTGAAGCCATTCCCTCCAATCAGTCTATGAATTCCGGTTCTTTGCGAAATAAGCTATT    500
Consensus                ttcaaaaccgaatctttgaagaaatggaggattatgacattcctgaagccattccctccaatcagtc atgaattccggttctttgcgaaataagctatt

Cla97C09G179080_1061_    TGGTGATGAATGGAATCTGCACCAATCAAAATCTGCAAGTACGTTGCAAGCCGAGTATTTATCTACTCGAGATATTGGGGTTCAGTTTAGTGCTGTGGAG    600
Cla97C09G179080_812_     TGGTGATGAATGGAATCTGCACCAATCAAAATCTGCAAGTACGTTGCAAGCCGAGTATTTATCTACTCGAGATATTGGGGTTCAGTTTAGTGCTGTGGAG    600
Consensus                tggtgatgaatggaatctgcaccaatcaaaatctgcaagtacgttgcaagccgagtatttatctactcgagatattggggttcagtttagtgctgtggag

Cla97C09G179080_1061_    GTTAAAAAATCTGCAGATGAGGTGAGATTCCAGAATTTGGGTGATGATGCAGCACACAGAATTAAAAAGAGTGGAGGGAAAAAGCGGAAAATTGAGTCTA    700
Cla97C09G179080_812_     GTTAAAAAATCTGCAGATGAGGTGAGATTCCAGAATTTGGGTGATGATGCAGCACACAGAATTAAAAAGAGTGGAGGGAAAAAGCGGAAAATTGAGTCTA    700
Consensus                gttaaaaaatctgcagatgaggtgagattccagaatttgggtgatgatgcagcacacagaattaaaaagagtggagggaaaaagcggaaaattgagtcta

Cla97C09G179080_1061_    GTGAGCACCATCCATCTGCTCATAATAGTGAAGATTTAGGAGATATTCGCTTTAGATTTTATGAAAGTGCTTCTGCTAGAAAGAGAACTGTGACAGCAGA    800
Cla97C09G179080_812_     GTGAGCACCATCCATCTGCTCATAATAGTGAAGATTTAGGAGATATTCGCTTTAGATTTTATGAAAGTGCTTCTGCTAGAAAGAGAACTGTGACAGCAGA    800
Consensus                gtgagcaccatccatctgctcataatagtgaagatttaggagatattcgctttagattttatgaaagtgcttctgctagaaagagaactgtgacagcaga

Cla97C09G179080_1061_    AGAAAGAGAAAGAGCTATCAACGCTGCAAAAGCTTTTGAGCCCGGTAATCCTTTCTGCAGAGTTGTCTTGCGACCATCCTATCTATACAGAGGTTGCATA    900
Cla97C09G179080_812_     AGAAAGAGAAAGAGCTATCAACGCTGCAAAAGCTTTTGAGCCCGGTAATCCTTTCTGCAGAGTTGTCTTGCGACCATCCTATCTATACAGAGGTTGCATA    900
Consensus                agaaagagaaagagctatcaacgctgcaaaagcttttgagcccggtaatcctttctgcagagttgtcttgcgaccatcctatctatacagaggttgcata

Cla97C09G179080_1061_    ATGTATTTGCCATCTTGCTTTGCTGAAAAGAATCTAAGTGGGGTTTCAGGATTCATCAAACTTCAGACACCTGACGGGAGACAGTGGCCAGTCCGATGTC   1000
Cla97C09G179080_812_     ATGTATTTGCCATCTTGCTTTGCTGAAAAGAATCTAAGTGGGGTTTCAGGATTCATCAAACTTCAGACACCTGACGGGAGACAGTGGCCAGTCCGATGTC   1000
Consensus                atgtatttgccatcttgctttgctgaaaagaatctaagtggggtttcaggattcatcaaacttcagacacctgacgggagacagtggccagtccgatgtc

Cla97C09G179080_1061_    TTTATAAAGTTGGTAGAGCGAAGTTAAGTCAGGGATGGTATGAGTTCTGCCTGGAAAATAATCTAGGGGAGGGTGACGTTTGTGTGTTTGAGCTTCTTAG   1100
Cla97C09G179080_812_     TTTATAAAGTTGGTAGAGCGAAGTTAAGTCAGGGATGGTATGAGTTCTGCCTGGAAAATAATCTAGGGGAGGGTGACGTTTGTGTGTTTGAGCTTCTTAG   1100
Consensus                tttataaagttggtagagcgaagttaagtcagggatggtatgagttctgcctggaaaataatctaggggagggtgacgtttgtgtgtttgagcttcttag

Cla97C09G179080_1061_    AATGAGAGAAATTGTGCTCAAAGTTACCATGTTTCGTGTCATTGAAGAAGGCGGACGAATGGCAACCCCCAATCCTGCTAGCATGATGAATCCACCTCCG   1200
Cla97C09G179080_812_     AATGAGAGAAATTGTGCTCAAAGTTACCATGTTTCGTGTCATTGAAGAAGGCGGACGAATGGCAACCCCCAATCCTGCTAGCATGATGAATCCACCTCCG   1200
Consensus                aatgagagaaattgtgctcaaagttaccatgtttcgtgtcattgaagaaggcggacgaatggcaacccccaatcctgctagcatgatgaatccacctccg

Cla97C09G179080_1061_    CTGCGAAGTGTTAGCCACATTAAACTGATCAGAAATTA                                                                 1238
Cla97C09G179080_812_     CTGCGAAGTGTTAGCCACATTAAACTGATCAGAAATTA                                                                 1238
Consensus                ctgcgaagtgttagccacattaaactgatcagaaatta
```

Figure S6A The result of *Cla97C09G179080* gene CDS sequence alignment

```
Cla97C09G179080_1061_    MTDFSAFRTGGFKRKMPRPHFHKLVLTSTIQARKLRIPETFVRMIRDELSAVATLTVPDGHVWRVGLRKADNKFWFEDGWQGFLEHYSIRVGYLLVFRYE    100
Cla97C09G179080_812_     MTDFSAFRTGGFKRKMPRPHFHKLVLTSTIQARKLRIPETFVRMIRDELSAVATLTVPDGHVWRVGLRKADNKFWFEDGWQGFLEHYSIRVGYLLVFRYE    100
Consensus                mtdfsafrtggfkrkmprphfhklvltstiqarklripetfvrmirdelsavatltvpdghvwrvglrkadnkfwfedgwqgflehysirvgyllvfrye

Cla97C09G179080_1061_    GNSAFCVFIFNLNASEINYQSAALSSNQRNNYSIQNRIFEEMEDYDIPEAIPSNQSMNSGSLRNKLFGDEWNLHQSKSASTLQAEYLSTRDIGVQFSAVE    200
Cla97C09G179080_812_     GNSAFCVFIFNLNASEINYQSAALSSNQRNNYSIQNRIFEEMEDYDIPEAIPSNQSMNSGSLRNKLFGDEWNLHQSKSASTLQAEYLSTRDIGVQFSAVE    200
Consensus                gnsafcvfifnlnaseinyqsaalssnqrnnysiqnrifeemedydipeaipsnqsmnsgslrnklfgdewnlhqsksastlqaeylstrdigvqfsave

Cla97C09G179080_1061_    VKKSADEVRFQNLGDDAAHRIKKSGGKKRKIESSEHHPSAHNSEDLGDIRFRFYESASARKRTVTAEERERAINAAKAFEPGNPFCRVVLRPSYLYRGCI    300
Cla97C09G179080_812_     VKKSADEVRFQNLGDDAAHRIKKSGGKKRKIESSEHHPSAHNSEDLGDIRFRFYESASARKRTVTAEERERAINAAKAFEPGNPFCRVVLRPSYLYRGCI    300
Consensus                vkksadevrfqnlgddaahrikksggkkrkiessehhpsahnsedlgdirfrfyesasarkrtvtaeererainaakafepgnpfcrvvlrpsylyrgci

Cla97C09G179080_1061_    MYLPSCFAEKNLSGVSGFIKLQTPDGRQWPVRCLYKVGRAKLSQGWYEFCLENNLGEGDVCVFELLRMREIVLKVTMFRVIEEGGRMATPNPASMMNPPP    400
Cla97C09G179080_812_     MYLPSCFAEKNLSGVSGFIKLQTPDGRQWPVRCLYKVGRAKLSQGWYEFCLENNLGEGDVCVFELLRMREIVLKVTMFRVIEEGGRMATPNPASMMNPPP    400
Consensus                mylpscfaeknlsgvsgfiklqtpdgrqwpvrclykvgraklsqgwyefclennlgegdvcvfellrmreivlkvtmfrvieeggrmatpnpasmmnppp

Cla97C09G179080_1061_    LRSVSHIKLIR                                                                                            411
Cla97C09G179080_812_     LRSVSHIKLIR                                                                                            411
Consensus                lrsvshiklir
```

Figure S6A-1 The result of *Cla97C09G179080* protein sequence alignment

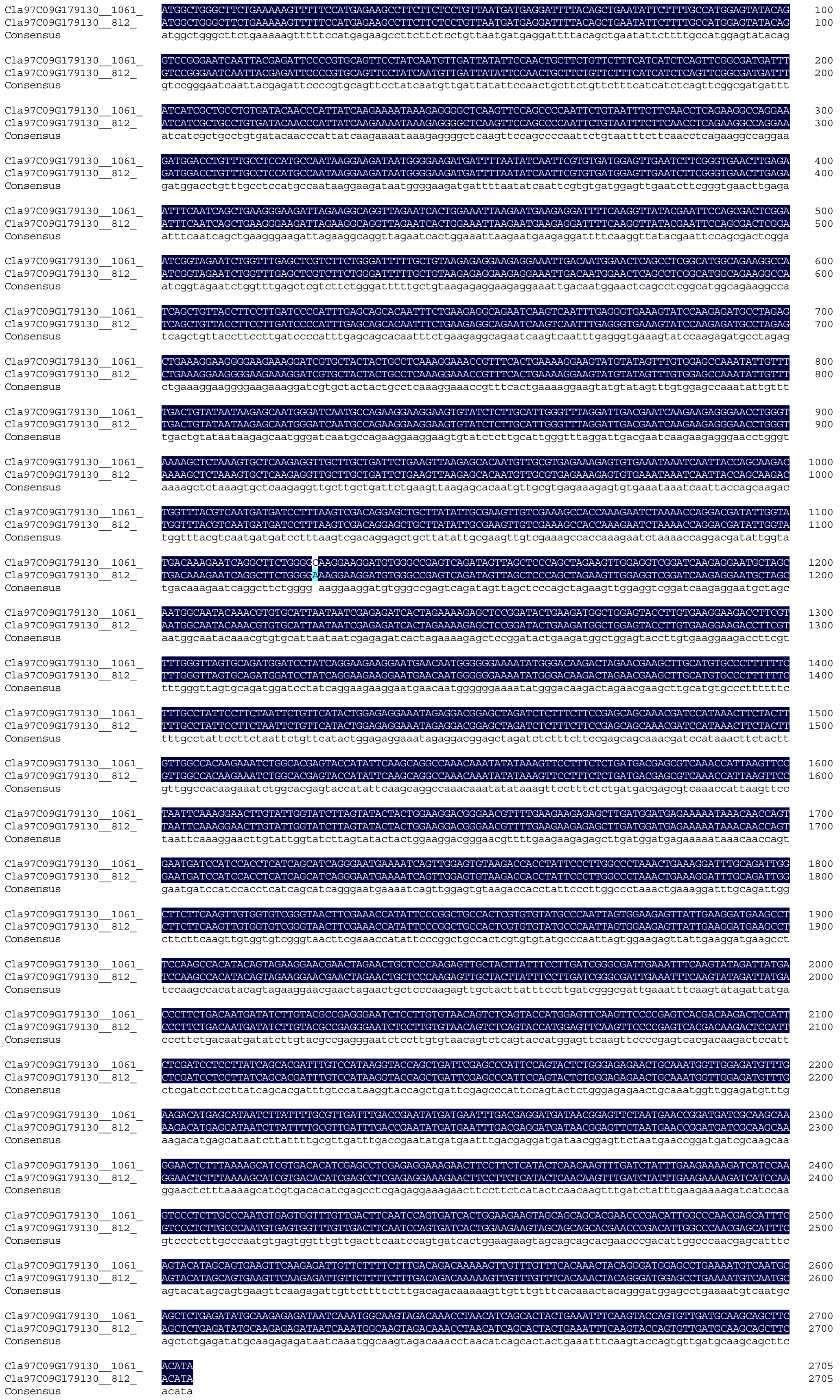

Figure S6B The result of *Cla97C09G179130* gene CDS sequence alignment

```
Cla97C09G179130__1061_    MAGLLKKFFHEKPSSPVNDEDFTAEYSFAMEYTGPGINYEIPRAVPINVDYIPTASVLSSSQFGDDLSSLPVIQPIIKKIKRGSSSSPNSVISSTSEGQE    100
Cla97C09G179130__812_     MAGLLKKFFHEKPSSPVNDEDFTAEYSFAMEYTGPGINYEIPRAVPINVDYIPTASVLSSSQFGDDLSSLPVIQPIIKKIKRGSSSSPNSVISSTSEGQE    100
Consensus                 magllkkffhekpsspvndedftaeysfameytgpginyeipravpinvdyiptasvlsssqfgddlsslpviqpiikkikrgsssspnsvisstsegqe

Cla97C09G179130__1061_    DGPVCLHANKEDNGEDDFNINSCDGVESSGELENFNQLKGRLEGRLESLEIKNEEDFQGYTNSSDSESVESGLSSSSGIFAVREEEEIDNGTQPRHGRRP    200
Cla97C09G179130__812_     DGPVCLHANKEDNGEDDFNINSCDGVESSGELENFNQLKGRLEGRLESLEIKNEEDFQGYTNSSDSESVESGLSSSSGIFAVREEEEIDNGTQPRHGRRP    200
Consensus                 dgpvclhankedngeddfninscdgvessgelenfnqlkgrlegrlesleikneedfqgytnssdsesvesglssssgifavreeeeidngtqprhgrrp

Cla97C09G179130__1061_    SAVTFLDPHLSSTISEEAESSQFEGESIQEMPRAERKGKKGSCYYCLKGNRFTEKEVCIVCGAKYCFDCIIRAMGSMPEGRKCISCIGFRIDESRRGNLG    300
Cla97C09G179130__812_     SAVTFLDPHLSSTISEEAESSQFEGESIQEMPRAERKGKKGSCYYCLKGNRFTEKEVCIVCGAKYCFDCIIRAMGSMPEGRKCISCIGFRIDESRRGNLG    300
Consensus                 savtfldphlsstiseeaessqfegesiqempraerkgkkgscyyclkgnrftekevcivcgakycfdciiramgsmpegrkciscigfridesrrgnlg

Cla97C09G179130__1061_    KSSKVLKRLLADSEVKSTMLREKECEINQLPARLVYVNDDPLSRQELLILRSCRKPPKNLKPGRYWYDKESGFWGKEGCGPSQIVSSQLEVGGRIKRNAS    400
Cla97C09G179130__812_     KSSKVLKRLLADSEVKSTMLREKECEINQLPARLVYVNDDPLSRQELLILRSCRKPPKNLKPGRYWYDKESGFWGKEGCGPSQIVSSQLEVGGRIKRNAS    400
Consensus                 ksskvlkrlladsevkstmlrekeceinqlparlvyvnddplsrqellilrscrkppknlkpgrywydkesgfwgkegcgpsqivssqlevggrikrnas

Cla97C09G179130__1061_    NGNTNVCINNREITRKELRILKMAGVPCEGRPSFWVSADGSYQEEGMNNGGKIWDKTRTKLACALFSLPIPSNSVHTGEEIEDGARSLSSEQQTIHKLLL    500
Cla97C09G179130__812_     NGNTNVCINNREITRKELRILKMAGVPCEGRPSFWVSADGSYQEEGMNNGGKIWDKTRTKLACALFSLPIPSNSVHTGEEIEDGARSLSSEQQTIHKLLL    500
Consensus                 ngntnvcinnreitrkelrilkmagvpcegrpsfwvsadgsyqeegmnnggkiwdktrtklacalfslpipsnsvhtgeeiedgarslsseqqtihklll

Cla97C09G179130__1061_    VGHKKSGTSTIFKQAKQIYKVPFSDDERQTIKFLIQRNLYWYLSILLEGRERFEEESLMDEKNKQPVNDPSTSSASGNENQLECKTTYSLGPKLKGFADW    600
Cla97C09G179130__812_     VGHKKSGTSTIFKQAKQIYKVPFSDDERQTIKFLIQRNLYWYLSILLEGRERFEEESLMDEKNKQPVNDPSTSSASGNENQLECKTTYSLGPKLKGFADW    600
Consensus                 vghkksgtstifkqakqiykvpfsdderqtikfliqrnlywylsillegrerfeeeslmdeknkqpvndpstssasgnenqleckttyslgpklkgfadw

Cla97C09G179130__1061_    LLQVVVSGNFETIFPAATRVYAQLVEELLKDEAFQATYSRRNELELLPRVATYFLDRAIEISSIDYDPSDNDILYAEGISLCNSLSTMEFKFPESRQDSI    700
Cla97C09G179130__812_     LLQVVVSGNFETIFPAATRVYAQLVEELLKDEAFQATYSRRNELELLPRVATYFLDRAIEISSIDYDPSDNDILYAEGISLCNSLSTMEFKFPESRQDSI    700
Consensus                 llqvvvsgnfetifpaatrvyaqlveellkdeafqatysrrnelellprvatyfldraieissidydpsdndilyaegislcnslstmefkfpesrqdsi

Cla97C09G179130__1061_    LDPPYQHDLSIRYQLIRAHSSTLGENCKWLEMFEDMSIILFCVDLTEYDEFDEDDNGVLMNRMIASKELFKSIVTHRASRGKNFLLILNKFDLFEEKIIQ    800
Cla97C09G179130__812_     LDPPYQHDLSIRYQLIRAHSSTLGENCKWLEMFEDMSIILFCVDLTEYDEFDEDDNGVLMNRMIASKELFKSIVTHRASRGKNFLLILNKFDLFEEKIIQ    800
Consensus                 ldppyqhdlsiryqlirahsstlgenckwlemfedmsiilfcvdlteydefdeddngvlmnrmiaskelfksivthrasrgknfllilnkfdlfeekiiq

Cla97C09G179130__1061_    VPLAQCEWFVDFNPVITGRSSSSTNPTLAQRAFQYIAVKFKRLFFSLTDKKLFVSQTTGMEPENVNAALRYAREIIKWQVDKPNISTTEISSTSVDASSF    900
Cla97C09G179130__812_     VPLAQCEWFVDFNPVITGRSSSSTNPTLAQRAFQYIAVKFKRLFFSLTDKKLFVSQTTGMEPENVNAALRYAREIIKWQVDKPNISTTEISSTSVDASSF    900
Consensus                 vplaqcewfvdfnpvitgrssssstnptlaqrafqyiavkfkrlffsltdkklfvsqttgmepenvnaalryareiikwqvdkpnistteisstsvdassf
```

Figure S6B-1 The result of *Cla97C09G179130* protein sequence alignment

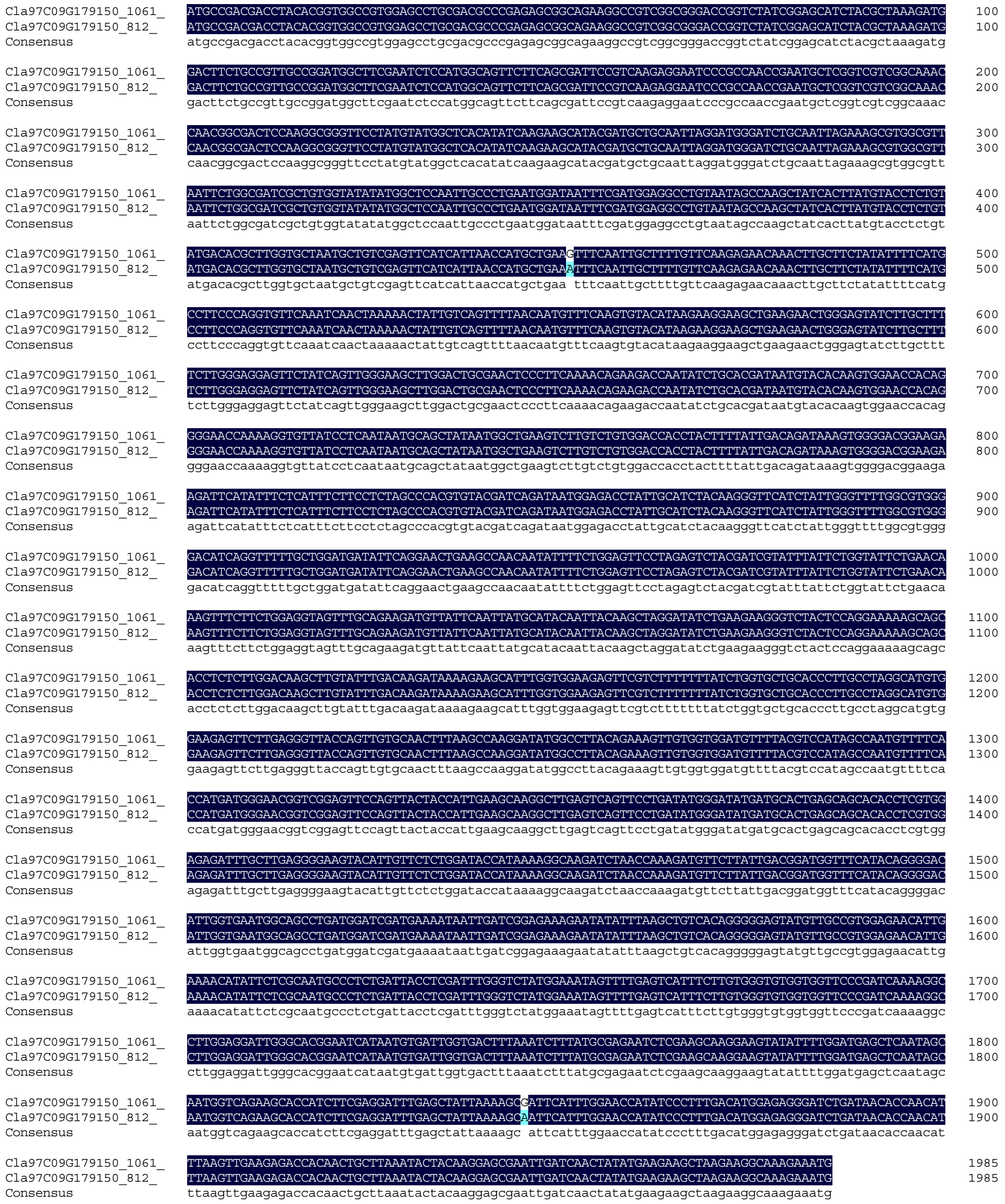

Figure S6C The result of *Cla97C09G179150* gene CDS sequence alignment

```
Cla97C09G179150_1061_   MPTTYTVAVEPATPESGRRPSAGPVYRSIYAKDGLLPLPDGFESPWQFFSDSVKRNPANRMLGRRQTNGDSKAGSYVWLTYQEAYDAAIRMGSAIRKRGV   100
Cla97C09G179150_812_    MPTTYTVAVEPATPESGRRPSAGPVYRSIYAKDGLLPLPDGFESPWQFFSDSVKRNPANRMLGRRQTNGDSKAGSYVWLTYQEAYDAAIRMGSAIRKRGV   100
Consensus               mpttytvavepatpesgrrpsagpvyrsiyakdgllplpdgfespwqffsdsvkrnpanrmlgrrqtngdskagsyvwltyqeaydaairmgsairkrgv

Cla97C09G179150_1061_   NSGDRCGIYGSNCPEWIISMEACNSQAITYVPLYDTLGANAVEFIINHAEVSIAFVQENKLASIFSCLPRCSNQLKTIVSFNNVSSVHKKEAEELGVSCF   200
Cla97C09G179150_812_    NSGDRCGIYGSNCPEWIISMEACNSQAITYVPLYDTLGANAVEFIINHAEISIAFVQENKLASIFSCLPRCSNQLKTIVSFNNVSSVHKKEAEELGVSCF   200
Consensus               nsgdrcgiygsncpewiismeacnsqaityvplydtlganavefiinhae siafvqenklasifsclprcsnqlktivsfnnvssvhkkeaeelgvscf

Cla97C09G179150_1061_   SWEEFYQLGSLDCELPSKQKTNICTIMYTSGTTGEPKGVILNNAAIMAEVLSVDHLLLLTDKVGTEEDSYFSFLPLAHVYDQIMETYCIYKGSSIGFWRG   300
Cla97C09G179150_812_    SWEEFYQLGSLDCELPSKQKTNICTIMYTSGTTGEPKGVILNNAAIMAEVLSVDHLLLLTDKVGTEEDSYFSFLPLAHVYDQIMETYCIYKGSSIGFWRG   300
Consensus               sweefyqlgsldcelpskqktnictimytsgttgepkgvilnnaaimaevlsvdhllllTdkvgteedsyfsflplahvydqimetyciykgssigfwrg

Cla97C09G179150_1061_   DIRFLLDDIQELKPTIFSGVPRVYDRIYSGILNKVSSGGSLQKMLFNYAYNYKLGYLKKGLLQEKAAPLLDKLVFDKIKEAFGGRVRLFLSGAAPLPRHV   400
Cla97C09G179150_812_    DIRFLLDDIQELKPTIFSGVPRVYDRIYSGILNKVSSGGSLQKMLFNYAYNYKLGYLKKGLLQEKAAPLLDKLVFDKIKEAFGGRVRLFLSGAAPLPRHV   400
Consensus               dirfllddiqelkptifsgvprvydriysgilnkvssggslqkmlfnyaynyklgylkkgllqekaaplldklvfdkikeafggrvrlflsgaaplprhv

Cla97C09G179150_1061_   EEFLRVTSCATLSQGYGLTESCGGCFTSIANVFTMMGTVGVPVTTIEARLESVPDMGYDALSSTPRGEICLRGSTLFSGYHKRQDLTKDVLIDGWFHTGD   500
Cla97C09G179150_812_    EEFLRVTSCATLSQGYGLTESCGGCFTSIANVFTMMGTVGVPVTTIEARLESVPDMGYDALSSTPRGEICLRGSTLFSGYHKRQDLTKDVLIDGWFHTGD   500
Consensus               eeflrvtscatlsqgygltescggcftsianvftmmgtvgvpvttiearlesvpdmgydalsstprgeiclrgstlfsgyhkrqdltkdvlidgwfhtgd

Cla97C09G179150_1061_   IGEWQPDGSMKIIDRRKNIFKLSQGEYVAVENIENIFSQCPLITSIWVYGNSFESFLVGVVVPDQKALEDWARNHNVIGDFKSLCENLEARKYILDELNS   600
Cla97C09G179150_812_    IGEWQPDGSMKIIDRRKNIFKLSQGEYVAVENIENIFSQCPLITSIWVYGNSFESFLVGVVVPDQKALEDWARNHNVIGDFKSLCENLEARKYILDELNS   600
Consensus               igewqpdgsmkiidrrknifklsqgeyvavenienifsqcplitsiwvygnsfesflvgvvvpdqkaledwarnhnvigdfkslcenlearkyildelns

Cla97C09G179150_1061_   NGQKHHLRGFELLKAIHLEPYPFDMERDLITPTFKLKRPQLLKYYKERIDQLYEEAKKAK                                          660
Cla97C09G179150_812_    NGQKHHLRGFELLKAIHLEPYPFDMERDLITPTFKLKRPQLLKYYKERIDQLYEEAKKAK                                          660
Consensus               ngqkhhlrgfellkaihlepypfdmerdlitptfklkrpqllkyykeridqlyeeakkak
```

Figure S6C-1 The result of *Cla97C09G179150* protein sequence alignment

```
Cla97C09G179220___1061_    ATGGGAAACAACCACGGCACCAAAAGGGGAAGCACGACGGTAGCCGCCATCAATCTCGACGCCTACGAAGCTGCGTGCCGGGCTGACGACGACGTCCGAT    100
Cla97C09G179220___812_     ATGGGAAACAACCACGGCACCAAAAGGGGAAGCACGACGGTAGCCGCCATCAATCTCGACGCCTACGAAGCTGCGTGCCGGGCTGACGACGACGTCCGAT    100
Consensus                  atgggaaacaaccacggcaccaaaaggggaagcacgacggtagccgccatcaatctcgacgcctacgaagctgcgtgccgggctgacgacgacgtccgat

Cla97C09G179220___1061_    CGTTCGACAAAGCCCTCCAAGCCAGAGCAAATCAAGTGCTGACGACATTAGCCGATGGGGGAGTGGAAGTCAGAGCTCTGTCTTTGGATTCCTTGAAACA    200
Cla97C09G179220___812_     CGTTCGACAAAGCCCTCCAAGCCAGAGCAAATCAAGTGCTGACGACATTAGCCGATGGGGGAGTGGAAGTCAGAGCTCTGTCTTTGGATTCCTTGAAACA    200
Consensus                  cgttcgacaaagccctccaagccagagcaaatcaagtgctgacgacattagccgatgggggagtggaagtcagagctctgtctttggattccttgaaaca

Cla97C09G179220___1061_    AGTGACGGAATGCTTGCTGGAAATGAACCAGGAGGTGGTTCGAGTGATATTGCAATGCAAAAAGGACATTTGGAAGAACCAGGAGCTGTTTGAGTTGGTG    300
Cla97C09G179220___812_     AGTGACGGAATGCTTGCTGGAAATGAACCAGGAGGTGGTTCGAGTGATATTGCAATGCAAAAAGGACATTTGGAAGAACCAGGAGCTGTTTGAGTTGGTG    300
Consensus                  agtgacggaatgcttgctggaaatgaaccaggaggtggttcgagtgatattgcaatgcaaaaaggacatttggaagaaccaggagctgtttgagttggtg

Cla97C09G179220___1061_    GAAGATTACTTTGAGAATAGCTTGGAGACTCTGGATTTCTGTACTGCTCTTGAAAACTGCCTGAAGCGAGCTCGGGACAGTCAGGTGCTGATTTTAATGG    400
Cla97C09G179220___812_     GAAGATTACTTTGAGAATAGCTTGGAGACTCTGGATTTCTGTACTGCTCTTGAAAACTGCCTGAAGCGAGCTCGGGACAGTCAGGTGCTGATTTTAATGG    400
Consensus                  gaagattactttgagaatagcttggagactctggatttctgtactgctcttgaaaactgcctgaagcgagctcgggacagtcaggtgctgattttaatgg

Cla97C09G179220___1061_    CGGTGAGGCAGTTCGAGGAGGAGGAAAGGGAAACCCAACTGGTTTCCAATCAGTTCGCTAAAACTCTGCAGGAATTGAAGAATTTTAAGGCCTCCGGCGA    500
Cla97C09G179220___812_     CGGTGAGGCAGTTCGAGGAGGAGGAAAGGGAAACCCAACTGGTTTCCAATCAGTTCGCTAAAACTCTGCAGGAATTGAAGAATTTTAAGGCCTCCGGCGA    500
Consensus                  cggtgaggcagttcgaggaggaggaaagggaaacccaactggtttccaatcagttcgctaaaactctgcaggaattgaagaattttaaggcctccggcga

Cla97C09G179220___1061_    CCCCTTCACCGATGATTTCTTCAAAATCTTCCACTCTGTTTATAAACACCAAACGGCCATGCTTGAGAAGCTTCAACAAAAGAAAAACAAACTTGACAAG    600
Cla97C09G179220___812_     CCCCTTCACCGATGATTTCTTCAAAATCTTCCACTCTGTTTATAAACACCAAACGGCCATGCTTGAGAAGCTTCAACAAAAGAAAAACAAACTTGACAAG    600
Consensus                  ccccttcaccgatgatttcttcaaaatcttccactctgtttataaacaccaaacggccatgcttgagaagcttcaacaaaagaaaaacaaacttgacaag

Cla97C09G179220___1061_    AAGCTAACGTCCATCAACACATGGAGGAAGCTCTCGTGCATGATATTCGCCGCCACATTTGCGGCGGTGTTAATATGCTCGGTGGTGGCAACGGTCATCA    700
Cla97C09G179220___812_     AAGCTAACGTCCATCAACACATGGAGGAAGCTCTCGTGCATGATATTCGCCGCCACATTCGCGGCGGTGTTAATATGCTCGGTGGTGGCAACGGTCATCA    700
Consensus                  aagctaacgtccatcaacacatggaggaagctctcgtgcatgatattcgccgccacatt gcggcggtgttaatatgctcggtggtggcaacggtcatca

Cla97C09G179220___1061_    CAGCACCACCCGTCGCAGCAGCCCTGTCGGCAGCTTCCTCCATTCCTGTAGGTTCCATGGGGAAATGGATTGATTCTCTATGGAAAAGCTATGAGAATGC    800
Cla97C09G179220___812_     CAGCACCACCCGTCGCAGCAGCCCTGTCGGCAGCTTCCTCCATTCCTGTAGGTTCCATGGGGAAATGGATTGATTCTCTATGGAAAAGCTATGAGAATGC    800
Consensus                  cagcaccacccgtcgcagcagccctgtcggcagcttcctccattcctgtaggttccatggggaaatggattgattctctatggaaaagctatgagaatgc

Cla97C09G179220___1061_    AGTGAAAGGGCAAAAGGAAGTGATAAACTCAATGCAAGTAGGGACTTACATTGCCATCAAAGACATGGACAACATTCGGATTTTGGTCGAAAAGCTCGAG    900
Cla97C09G179220___812_     AGTGAAAGGGCAAAAGGAAGTGATAAACTCAATGCAAGTAGGGACTTACATTGCCATCAAAGACATGGACAACATTCGGATTTTGGTCGAAAAGCTCGAG    900
Consensus                  agtgaaagggcaaaaggaagtgataaactcaatgcaagtagggacttacattgccatcaaagacatggacaacattcggattttggtcgaaaagctcgag

Cla97C09G179220___1061_    ATCGAGATTGAAGGAATGTTGGAGAAGGCAGATTTTGCCATTAAAGAGGAAGCTTTGAAGGTTGGGGTGGAAGAGATGAAGAAGAAACTTGGGGTGTTCA   1000
Cla97C09G179220___812_     ATCGAGATTGAAGGAATGTTGGAGAAGGCAGATTTTGCCATTAAAGAGGAAGCTTTGAAGGTTGGGGTGGAAGAGATGAAGAAGAAACTTGGGGTGTTCA   1000
Consensus                  atcgagattgaaggaatgttggagaaggcagattttgccattaaagaggaagctttgaaggttggggtggaagagatgaagaagaaacttggggtgttca

Cla97C09G179220___1061_    TGAAAACTGTTGAGGATTTAGGGGTTCAAGCTGATTTGTGTAGTAGAGACATTACAAGGGCAAGGACTGTGGTTTTGCAAAGAATTATAAAACATCCCAA   1100
Cla97C09G179220___812_     TGAAAACTGTTGAGGATTTAGGGGTTCAAGCTGATTTGTGTAGTAGAGACATTACAAGGGCAAGGACTGTGGTTTTGCAAAGAATTATAAAACATCCCAA   1100
Consensus                  tgaaaactgttgaggatttaggggttcaagctgatttgtgtagtagagacattacaagggcaaggactgtggttttgcaaagaattataaaacatcccaa

Cla97C09G179220___1061_    TTATTA                                                                                                 1106
Cla97C09G179220___812_     TTATTA                                                                                                 1106
Consensus                  ttatta
```

Figure S6D The result of *Cla97C09G179220* gene CDS sequence alignment

```
Cla97C09G179220___1061_    MGNNHGTKRGSTTVAAINLDAYEAACRADDDVRSFDKALQARANQVLTTLADGGVEVRALSLDSLKQVTECLLEMNQEVVRVILQCKKDIWKNQELFELV    100
Cla97C09G179220___812_     MGNNHGTKRGSTTVAAINLDAYEAACRADDDVRSFDKALQARANQVLTTLADGGVEVRALSLDSLKQVTECLLEMNQEVVRVILQCKKDIWKNQELFELV    100
Consensus                  mgnnhgtkrgsttvaainldayeaacradddvrsfdkalqaranqvlttladggvevralsldslkqvtecllemnqevvrvilqckkdiwknqelfelv

Cla97C09G179220___1061_    EDYFENSLETLDFCTALENCLKRARDSQVLILMAVRQFEEEERETQLVSNQFAKTLQELKNFKASGDPFTDDFFKIFHSVYKHQTAMLEKLQQKKNKLDK    200
Cla97C09G179220___812_     EDYFENSLETLDFCTALENCLKRARDSQVLILMAVRQFEEEERETQLVSNQFAKTLQELKNFKASGDPFTDDFFKIFHSVYKHQTAMLEKLQQKKNKLDK    200
Consensus                  edyfensletldfctalenclkrardsqvlilmavrqfeeeeretqlvsnqfaktlqelknfkasgdpftddffkifhsvykhqtamleklqqkknkldk

Cla97C09G179220___1061_    KLTSINTWRKLSCMIFAATFAAVLICSVVATVITAPPVAAALSAASSIPVGSMGKWIDSLWKSYENAVKGQKEVINSMQVGTYIAIKDMDNIRILVEKLE    300
Cla97C09G179220___812_     KLTSINTWRKLSCMIFAATFAAVLICSVVATVITAPPVAAALSAASSIPVGSMGKWIDSLWKSYENAVKGQKEVINSMQVGTYIAIKDMDNIRILVEKLE    300
Consensus                  kltsintwrklscmifaatfaavlicsvvatvitappvaaalsaassipvgsmgkwidslwksyenavkgqkevinsmqvgtyiaikdmdnirilvekle

Cla97C09G179220___1061_    IEIEGMLEKADFAIKEEALKVGVEEMKKKLGVFMKTVEDLGVQADLCSRDITRARTVVLQRIIKHPN                                    367
Cla97C09G179220___812_     IEIEGMLEKADFAIKEEALKVGVEEMKKKLGVFMKTVEDLGVQADLCSRDITRARTVVLQRIIKHPN                                    367
Consensus                  ieiegmlekadfaikeealkvgveemkkklgvfmktvedlgvqadlcsrditrartvvlqriikhpn
```

Figure S6D-1 The result of *Cla97C09G179220* protein sequence alignment

```
Cla97C09G179350__1061_   ATGGCAAGAAAGAGAAAGGCTGTTGAAGGAGTGGAAGACAAGAGCTCAAGTGAAGGAGGAGCTATGGCTTGGGATGAGATGGTGAAGGAGGCAGCGGCAA   100
Cla97C09G179350__812_    ATGGCAAGAAAGAGAAAGGCTGTTGAAGGAGTGGAAGACAAGAGCTCAAGTGAAGGAGGAGCTATGGCTTGGGATGAGATGGTGAAGGAGGCAGCGGCAA   100
Consensus                atggcaagaaagagaaaggctgttgaaggagtggaagacaagagctcaagtgaaggaggagctatggcttgggatgagatggtgaaggaggcagcggcaa

Cla97C09G179350__1061_   CAGCAGCAGCGCTTGGCGGAGGCGTTCGGAGGGCCCGAAAGCGATTTGTTGGTGTTCGACAACGACCATCAGGGCGATGGGTGGCGGAGATCAAGGACAC   200
Cla97C09G179350__812_    CAGCAGCAGCGCTTGGCGGAGGCGTTCGGAGGGCCCGAAAGCGATTTGTTGGTGTTCGACAACGACCATCAGGGCGATGGGTGGCGGAGATCAAGGACAC   200
Consensus                cagcagcagcgcttggcggaggcgttcggagggcccgaaagcgatttgttggtgttcgacaacgaccatcagggcgatgggtggcggagatcaaggacac

Cla97C09G179350__1061_   TATACAGAAAATCAGAGTATGGTTAGGGACATTTGACACAGCGGAGGAAGCAGCCAGAGCTTACGATGAGGCGGCTTGTTTACTCCGTGGCTCCAACACA   300
Cla97C09G179350__812_    TATACAGAAAATCAGAGTATGGTTAGGGACATTTGACACAGCGGAGGAAGCAGCCAGAGCTTACGATGAGGCGGCTTGTTTACTCCGTGGCTCCAACACA   300
Consensus                tatacagaaaatcagagtatggttagggacatttgacacagcggaggaagcagccagagcttacgatgaggcggcttgtttactccgtggctccaacaca

Cla97C09G179350__1061_   AGAACAAACTTCTGGCCTTGCTCCCCTTTATCATCTTCATCACCAGCCCTTCCTTCAAAGATCACCAATTTGCTTATCCAAAGGCTTAATGCAAGAAACA   400
Cla97C09G179350__812_    AGAACAAACTTCTGGCCTTGCTCCCCTTTATCATCTTCATCACCAGCCCTTCCTTCAAAGATCACCAATTTGCTTATCCAAAGGCTTAATGCAAGAAACA   400
Consensus                agaacaaacttctggccttgctcccctttatcatcttcatcaccagcccttccttcaaagatcaccaatttgcttatccaaaggcttaatgcaagaaaca

Cla97C09G179350__1061_   ATAACTCCTCCATCAATAATCTTCCAAACAATCAACAAGAACAAAAACATCAGCCAGCTCAAGAGAGGATGTTACACCCAAATGAACATGAAAGCAGAGA   500
Cla97C09G179350__812_    ATAACTCCTCCATCAATAATCTTCCAAACAATCAACAAGAACAAAAACATCAGCCAGCTCAAGAGAGGATGTTACACCCAAATGAACATGAAAGCAGAGA   500
Consensus                ataactcctccatcaataatcttccaaacaatcaacaagaacaaaaacatcagccagctcaagagaggatgttacacccaaatgaacatgaaagcagaga

Cla97C09G179350__1061_   AGAGTTAGCAACAACATGTTTCACAGACAGAGTTCTAAGTGATTTGCTCAATGACCAAGAAGTTTTCCCCACTAATCCCAATATTGAAGAAATCAGTAGG   600
Cla97C09G179350__812_    AGAGTTAGCAACAACATGTTTCACAGACAGAGTTCTAAGTGATTTGCTCAATGACCAAGAAGTTTTCCCCACTAATCCCAATATTGAAGAAATCAGTAGG   600
Consensus                agagttagcaacaacatgtttcacagacagagttctaagtgatttgctcaatgaccaagaagttttccccactaatcccaatattgaagaaatcagtagg

Cla97C09G179350__1061_   AGTTTTGAGTCATGTTTGACTGAGAAAGATGAATCTGATAGTGGAGAAATGTATAGCAGCAACTGGATGGGGATGACCCAGATGAATAACTCAGATGGGG   700
Cla97C09G179350__812_    AGTTTTGAGTCATGTTTGACTGAGAAAGATGAATCTGATAGTGGAGAAATGTATAGCAGCAACTGGATGGGGATGACCCAGATGAATAACTCAGATGGGG   700
Consensus                agttttgagtcatgtttgactgagaaagatgaatctgatagtggagaaatgtatagcagcaactggatggggatgacccagatgaataactcagatgggg

Cla97C09G179350__1061_   GAGATGAAAGAAATGGATTAGTTCAACAACAAGAAGAAGAAGAAGAATCAAATGTTTTGGATTTCCATTTCCTTGATAACATTGGGCCACCATGTTACTA   800
Cla97C09G179350__812_    GAGATGAAAGAAATGGATTAGTTCAACAAGAAGAAGAAGAAGAAGAATCAAATGTTTTGGATTTCCATTTCCTTGATAACATTGGGCCACCATGTTACTA   800
Consensus                gagatgaaagaaatggattagttcaacaa aagaagaagaagaagaatcaaatgttttggatttccatttccttgataacattgggccaccatgttacta

Cla97C09G179350__1061_   CTCTCCATTTGAGATTGCAGAAGAGATTGGTGAGCCAATGGAGGGAGGAGAAGGGAGTGAAGATGAGCCATCTTCAATGCTAAGAGAAGCTATGAAAAGG   900
Cla97C09G179350__812_    CTCTCCATTTGAGATTGCAGAAGAGATTGGTGAGCCAATGGAGGGAGGAGAAGGGAGTGAAGATGAGCCATCTTCAATGCTAAGAGAAGCTATGAAAAGG   900
Consensus                ctctccatttgagattgcagaagagattggtgagccaatggagggaggagaagggagtgaagatgagccatcttcaatgctaagagaagctatgaaaagg

Cla97C09G179350__1061_   ATGAAATATGAGAGGAAAATTTCAGCTTCTCTGTATGCTTTCAATGGAATACCAGAGTGCTTAAAATTGAAGCTTGGAGAAGGAAGTGAGAATGAAAGAG  1000
Cla97C09G179350__812_    ATGAAATATGAGAGGAAAATTTCAGCTTCTCTGTATGCTTTCAATGGAATACCAGAGTGCTTAAAATTGAAGCTTGGAGAAGGAAGTGAGAATGAAAGAG  1000
Consensus                atgaaatatgagaggaaaatttcagcttctctgtatgctttcaatggaataccagagtgcttaaaattgaagcttggagaaggaagtgagaatgaaagag

Cla97C09G179350__1061_   GGAGATCTAATTCAGAGCTAATAACAAGCTTGAGAAAAGCATGTGACAGAAGAAGATCAGATGAGGAGAAGGTTGAAGAAGAAGAAGAAGAAGAAGAAGA  1100
Cla97C09G179350__812_    GGAGATCTAATTCAGAGCTAATAACAAGCTTGAGAAAAGCATGTGACAGAAGAAGATCAGATGAGGAGAAGGTTGAAGAAGAAGAAGAAGAAGAAGAAGA  1100
Consensus                ggagatctaattcagagctaataacaagcttgagaaaagcatgtgacagaagaagatcagatgaggagaaggttgaagaagaagaagaagaagaagaaga

Cla97C09G179350__1061_   AGAAGAAGAGTATCAGAAGAAAATGGAGGGAGATAAAGAAGAAACTTGTTCAGCAGAAATGAAGTCTTTTTCAAGCACTGAAGTTGACTTGTCCATTTGG  1200
Cla97C09G179350__812_    AGAAGAAGAGTATCAGAAGAAAATGGAGGGAGATAAAGAAGAAACTTGTTCAGCAGAAATGAAGTCTTTTTCAAGCACTGAAGTTGACTTGTCCATTTGG  1200
Consensus                agaagaagagtatcagaagaaaatggagggagataaagaagaaacttgttcagcagaaatgaagtctttttcaagcactgaagttgacttgtccatttgg

Cla97C09G179350__1061_   AGTTCACTAGATCTTCAACCCATTTGCTTTGTTAACTG                                                                1238
Cla97C09G179350__812_    AGTTCACTAGATCTTCAACCCATTTGCTTTGTTAACTG                                                                1238
Consensus                agttcactagatcttcaacccatttgctttgttaactg
```

Figure S6E The result of *Cla97C09G179350* gene CDS sequence alignment

```
Cla97C09G179350__1061_   MARKRKAVEGVEDKSSSEGGAMAWDEMVKEAAATAAALGGGVRRARKRFVGVRQRPSGRWVAEIKDTIQKIRVWLGTFDTAEEAARAYDEAACLLRGSNT   100
Cla97C09G179350__812_    MARKRKAVEGVEDKSSSEGGAMAWDEMVKEAAATAAALGGGVRRARKRFVGVRQRPSGRWVAEIKDTIQKIRVWLGTFDTAEEAARAYDEAACLLRGSNT   100
Consensus                markrkavegvedksssseggamawdemvkeaaataaalgggvrrarkrfvgvrqrpsgrwvaeikdtiqkirvwlgtfdtaeeaaraydeaacllrgsnt

Cla97C09G179350__1061_   RTNFWPCSPLSSSSPALPSKITNLLIQRLNARNNNSSINNLPNNQQEQKHQPAQERMLHPNEHESREELATTCFTDRVLSDLLNDQEVFPTNPNIEEISR   200
Cla97C09G179350__812_    RTNFWPCSPLSSSSPALPSKITNLLIQRLNARNNNSSINNLPNNQQEQKHQPAQERMLHPNEHESREELATTCFTDRVLSDLLNDQEVFPTNPNIEEISR   200
Consensus                rtnfwpcsplsssspalpskitnlliqrlnarnnnssinnlpnnqqeqkhqpaqermlhpnehesreelattcftdrvlsdllndqevfptnpnieeisr

Cla97C09G179350__1061_   SFESCLTEKDESDSGEMYSSNWMGMTQMNNSDGGDERNGLVQQQEEEEESNVLDFHFLDNIGPPCYYSPFEIAEEIGEPMEGGEGSEDEPSSMLREAMKR   300
Cla97C09G179350__812_    SFESCLTEKDESDSGEMYSSNWMGMTQMNNSDGGDERNGLVQQEEEEEESNVLDFHFLDNIGPPCYYSPFEIAEEIGEPMEGGEGSEDEPSSMLREAMKR   300
Consensus                sfescltekdesdsgemyssnwmgmtqmnnsdggdernglvqq eeeeesnvldfhfldnigppcyyspfeiaeeigepmeggegsedepssmlreamkr

Cla97C09G179350__1061_   MKYERKISASLYAFNGIPECLKLKLGEGSENERGRSNSELITSLRKACDRRRSDEEKVEEEEEEEEEEEEYQKKMEGDKEETCSAEMKSFSSTEVDLSIW   400
Cla97C09G179350__812_    MKYERKISASLYAFNGIPECLKLKLGEGSENERGRSNSELITSLRKACDRRRSDEEKVEEEEEEEEEEEEYQKKMEGDKEETCSAEMKSFSSTEVDLSIW   400
Consensus                mkyerkisaslyafngipeclklklgegsenergrsnselitslrkacdrrrsdeekveeeeeeeeeeeeyqkkmegdkeetcsaemksfsstevdlsiw

Cla97C09G179350__1061_   SSLDLQPICFV                                                                                           411
Cla97C09G179350__812_    SSLDLQPICFV                                                                                           411
Consensus                ssldlqpicfv
```

Figure S6E-1 The result of *Cla97C09G179350* protein sequence alignment

```
Cla97C09G180040__1061_   ATGGCGTTGGAAGAATTATCATTCTTCACTTTCACTCTTCTCCTTCTTACCTTCTTCCCTTTAATATCATCTTCTCAGCCAAACTCTGCACATTCCCATG   100
Cla97C09G180040__812_    ATGGCGTTGGAAGAATTATCATTCTTCACTTTCACTCTTCTCCTTCTTACCTTCTTCCCTTTAATATCATCTTCTCAGCCAAACTCTGCACATTCCCATG   100
Consensus                atggcgttggaagaattatcattcttcactttcactcttctccttcttaccttcttccctttaatatcatcttctcagccaaactctgcacattcccatg

Cla97C09G180040__1061_   GAAAAACACCATCACAATTTGTGTTTCTGAACCAACTTCATGGATCCCAAAAGGGTGACAAAGTTGAAGGTATCCACCAACTGAAGAAATACCTTCAGCA   200
Cla97C09G180040__812_    GAAAAACACCATCACAATTTGTGTTTCTGAACCAACTTCATGGATCCCAAAAGGGTGACAAAGTTGAAGGTATCCACCAACTGAAGAAATACCTTCAGCA   200
Consensus                gaaaaacaccatcacaatttgtgtttctgaaccaacttcatggatcccaaaagggtgacaaagttgaaggtatccaccaactgaagaaataccttcagca

Cla97C09G180040__1061_   ATTCGGCTACTTGAACGATGCTCAAATTCATTCCAAAAATAATGATAACGAGTTTGATGAGTTGTTAGAGTCTACCATTAAAACGTACCAAAAGAATTAC   300
Cla97C09G180040__812_    ATTCGGCTACTTGAACGATGCTCAAATTCATTCCAAAAATAATGATAACGAGTTTGATGAGTTGTTAGAGTCTACCATTAAAACGTACCAAAAGAATTAC   300
Consensus                attcggctacttgaacgatgctcaaattcattccaaaaataatgataacgagtttgatgagttgttagagtctaccattaaaacgtaccaaaagaattac

Cla97C09G180040__1061_   AATCTCAAAGTCACTGGGACTCTTGATGCCATGACGATAGCTCAAATGTCAAAGCCTCGATGTGGTGTTGCGGATATCATCCATGGCAATACTTGGATGA   400
Cla97C09G180040__812_    AATCTCAAAGTCACTGGGACTCTTGATGCCATGACGATAGCTCAAATGTCAAAGCCTCGATGTGGTGTTGCGGATATCATCCATGGCAATACTTGGATGA   400
Consensus                aatctcaaagtcactgggactcttgatgccatgacgatagctcaaatgtcaaagcctcgatgtggtgttgcggatatcatccatggcaatacttggatga

Cla97C09G180040__1061_   GATCAAGCAAAAAAAGAAAACAACTTGAACATGGAATTGGACATTTTCATAGGGTTTCTCACTTTGCTTTCTTTGAAGGAAATCCCAAGTGGCCTGCTGC   500
Cla97C09G180040__812_    GATCAAGCAAAAAAAGAAAACAACTTGAACATGGAATTGGACATTTTCATAGGGTTTCTCACTTTGCTTTCTTTGAAGGAAATCCCAAGTGGCCTGCTGC   500
Consensus                gatcaagcaaaaaaagaaaacaacttgaacatggaattggacattttcatagggtttctcactttgctttctttgaaggaaatcccaagtggcctgctgc

Cla97C09G180040__1061_   TAAATCTCACCTCACTTATGGGTTCGTTCCAGGAACTCCACCGGAAACAGTAAGTCCGGTAGCCCGAGCTTTTGCTACTTGGGCGGCAAACTCTCACTTC   600
Cla97C09G180040__812_    TAAATCTCACCTCACTTATGGGTTCGTTCCAGGAACTCCACCGGAAACAGTAAGTCCGGTAGCCCGAGCTTTTGCTACTTGGGCGGCAAACTCTCACTTC   600
Consensus                taaatctcacctcacttatgggttcgttccaggaactccaccggaaacagtaagtccggtagcccgagcttttgctacttgggcggcaaactctcacttc

Cla97C09G180040__1061_   ACCTTTTCCCAAGCTTTGGACAACCAAACTTCAGACATCAAAATAGGGTTCGAAAGTGGTGATCATGGAGATGGATATCCATTCGATGGTGTTGGAGGAG   700
Cla97C09G180040__812_    ACCTTTTCCCAAGCTTTGGACAACCAAACTTCAGACATCAAAATAGGGTTCGAAAGTGGTGATCATGGAGATGGATATCCATTCGATGGTGTTGGAGGAG   700
Consensus                accttttcccaagctttggacaaccaaacttcagacatcaaaatagggttcgaaagtggtgatcatggagatggatatccattcgatggtgttggaggag

Cla97C09G180040__1061_   TGATAGCCCATGCTTTTTCACCGCCGGATGGCCGGTTTCATCTCGATGCCGAGGAGTCTTGGGTGGCCGGTGTCATCTCTGGTTCGTTTGATTTGGAGAC   800
Cla97C09G180040__812_    TGATAGCCCATGCTTTTTCACCGCCGGATGGCCGGTTTCATCTCGATGCCGAGGAGTCTTGGGCGGCCGGTGTCATCTCTGGTTCGTTTGATTTGGAGAC   800
Consensus                tgatagcccatgctttttcaccgccggatggccggtttcatctcgatgccgaggagtcttggg ggccggtgtcatctctggttcgtttgatttggagac

Cla97C09G180040__1061_   GGTGGCATTGCATGAGATTGGACACCTTCTAGGCCTTCAACATAGCTCCATTGAAGGGGCTATCATGTGGCCATCAGTCCCGGAAGGAGCTTCCAAGGGT   900
Cla97C09G180040__812_    GGTGGCATTGCATGAGATTGGACACCTTCTAGGCCTTCAACATAGCTCCATTGAAGGGGCTATCATGTGGCCATCAGTCCCGGAAGGAGCTTCCAAGGGT   900
Consensus                ggtggcattgcatgagattggacaccttctaggccttcaacatagctccattgaaggggctatcatgtggccatcagtcccggaaggagcttccaagggt

Cla97C09G180040__1061_   TTGCATGCAGATGATATTGCAGGCATTAAGGCTTTATATAATAACACTCTCTA                                                 953
Cla97C09G180040__812_    TTGCATGCAGATGATATTGCAGGCATTAAGGCTTTATATAATAACACTCTCTA                                                 953
Consensus                ttgcatgcagatgatattgcaggcattaaggctttatataataacactctcta
```

Figure S6F The result of *Cla97C09G180040* gene CDS sequence alignment

```
Cla97C09G180040__1061_   MALEELSFFTFTLLLLTFFPLISSSQPNSAHSHGKTPSQFVFLNQLHGSQKGDKVEGIHQLKKYLQQFGYLNDAQIHSKNNDNEFDELLESTIKTYQKNY   100
Cla97C09G180040__812_    MALEELSFFTFTLLLLTFFPLISSSQPNSAHSHGKTPSQFVFLNQLHGSQKGDKVEGIHQLKKYLQQFGYLNDAQIHSKNNDNEFDELLESTIKTYQKNY   100
Consensus                maleelsfftftlllltffplisssqpnsahshgktpsqfvflnqlhgsqkgdkvegihqlkkylqqfgylndaqihsknndnefdellestiktyqkny

Cla97C09G180040__1061_   NLKVTGTLDAMTIAQMSKPRCGVADIIHGNTWMRSSKKRKQLEHGIGHFHRVSHFAFFEGNPKWPAAKSHLTYGFVPGTPPETVSPVARAFATWAANSHF   200
Cla97C09G180040__812_    NLKVTGTLDAMTIAQMSKPRCGVADIIHGNTWMRSSKKRKQLEHGIGHFHRVSHFAFFEGNPKWPAAKSHLTYGFVPGTPPETVSPVARAFATWAANSHF   200
Consensus                nlkvtgtldamtiaqmskprcgvadiihgntwmrsskkrkqlehgighfhrvshfaffegnpkwpaakshltygfvpgtppetvspvarafatwaanshf

Cla97C09G180040__1061_   TFSQALDNQTSDIKIGFESGDHGDGYPFDGVGGVIAHAFSPPDGRFHLDAEESWVAGVISGSFDLETVALHEIGHLLGLQHSSIEGAIMWPSVPEGASKG   300
Cla97C09G180040__812_    TFSQALDNQTSDIKIGFESGDHGDGYPFDGVGGVIAHAFSPPDGRFHLDAEESWAAGVISGSFDLETVALHEIGHLLGLQHSSIEGAIMWPSVPEGASKG   300
Consensus                tfsqaldnqtsdikigfesgdhgdgypfdgvggviahafsppdgrfhldaeesw agvisgsfdletvalheighllglqhssiegaimwpsvpegaskg

Cla97C09G180040__1061_   LHADDIAGIKALYNNT                                                                                      316
Cla97C09G180040__812_    LHADDIAGIKALYNNT                                                                                      316
Consensus                lhaddiagikalynnt
```

Figure S6F-1 The result of *Cla97C09G180040* protein sequence alignment

```
Cla97C09G180070__1061_    ATGGATTTCAAATCATCGTCTCTCCAACTCTTCCTACTTCTTCTTGCCTCCATAGCATTATTTCACCCGATTTCGACACATGATCTCGATCACATCCATA    100
Cla97C09G180070__812_     ATGGATTTCAAATCATCGTCTCTCCAACTCTTCCTACTTCTTCTTGCCTCCATAGCATTATTTCACCCGATTTCGACACATGATCTCGATCACATCCATA    100
Consensus                 atggatttcaaatcatcgtctctccaactcttcctacttcttcttgcctccatagcattatttcacccgatttcgacacatgatctcgatcacatccata

Cla97C09G180070__1061_    AATCATCTCACTTTCTATTTCCTCAACATCTTCTGGGAAGTCGTAAGGATCACAACATCGAAGGAATCCATAGCTTAAGAAAAAATTATAACATTATCGA    200
Cla97C09G180070__812_     AATCATCTCACTTTCTATTTCCTCAACATCTTCTGGGAAGTCGTAAGGATCACAACATCGAAGGAATCCATAGCTTAAGAAAAAATTATAACATTATCGA    200
Consensus                 aatcatctcactttctatttcctcaacatcttctgggaagtcgtaaggatcacaacatcgaaggaatccatagcttaagaaaaaattataacattatcga

Cla97C09G180070__1061_    TACCAATGGCGCTCATAATAACACCTTCGACCACCACCTAGAATCCGCCGTAAAAAAATACCAAAAATTCTTCAAGCTTAACGAGAGTGGAATTTTAGAC    300
Cla97C09G180070__812_     TACCAATGGCGCTCATAATAACACCTTCGACCACCACCTAGAATCCGCCGTAAAAAAATACCAAAAATTCTTCAAGCTTAACGAGAGTGGAATTTTAGAC    300
Consensus                 taccaatggcgctcataataacaccttcgaccaccacctagaatccgccgtaaaaaaataccaaaaattcttcaagcttaacgagagtggaattttagac

Cla97C09G180070__1061_    GTGGAGACATTGTACCAAATGTCAGAGTCCCGTTGTTCGGTTCCCGACATATTCGAGAAGGACGACAATGAGACGAGTAAACTCCACATAGGAAGCAAGT    400
Cla97C09G180070__812_     GTGGAGACATTGTACCAAATGTCAGAGTCCCGTTGTTCGGTTCCCGACATATTCGAGAAGGACGACAATGAGACGAGTAAACTCCACATAGGAAGCAAGT    400
Consensus                 gtggagacattgtaccaaatgtcagagtcccgttgttcggttcccgacatattcgagaaggacgacaatgagacgagtaaactccacataggaagcaagt

Cla97C09G180070__1061_    ACACATTTTTTCCCGGGAGAATAAAATGGGCGAGTTGGAAGAAATACCAATTAAAATACTCATTCATTCGGAATTTCCCAGAAGAGTTTAAGGAGTCGGT    500
Cla97C09G180070__812_     ACACATTTTTTCCCGGGAGAATAAAATGGGCGAGTTGGAAGAAATACCAATTAAAATACTCATTCATTCGGAATTTCCCAGAAGAGTTTAAGGAGTCGGT    500
Consensus                 acacattttttcccgggagaataaaatgggcgagttggaagaaataccaattaaaatactcattcattcggaatttcccagaagagtttaaggagtcggt

Cla97C09G180070__1061_    GAGTGCGGCGTTTATGATATGGTATGAACGCAGCCGATTTAATTTCACAGAAGTTGTTGAGAATGAAGATGCGGATATAAGAATAAGCTTTGAGGTAGGA    600
Cla97C09G180070__812_     GAGTGCGGCGTTTATGATATGGTATGAACGCAGCCGATTTAATTTCACAGAAGTTGTTGAGAATGAAGATGCGGATATAAGAATAAGCTTTGAGGTAGGA    600
Consensus                 gagtgcggcgtttatgatatggtatgaacgcagccgatttaatttcacagaagttgttgagaatgaagatgcggatataagaataagctttgaggtagga

Cla97C09G180070__1061_    AACCATGGAGATTTGCATCCTTTCACGAAGGAAGTTTTGGCACATACGTTTGGGCCTGGGGATGGGAGATTTCACTTCAATGCTGAACAATCTTTTTCTG    700
Cla97C09G180070__812_     AACCATGGAGATTTGCATCCTTTCACGAAGGAAGTTTTGGCACATACGTTTGGGCCTGGGGATGGGAGATTTCACTTCAATGCTGAACAATCTTTTTCTG    700
Consensus                 aaccatggagatttgcatcctttcacgaaggaagttttggcacatacgtttgggcctggggatgggagatttcacttcaatgctgaacaatctttttctg

Cla97C09G180070__1061_    TTGAAGTTACATATGGTAAGTATCATGTGAGAACTTTGGCACTTCATGAGCTCGGACATGCACTTGGGCTGGCGCACAGCACCAATGAAGATGCTATCAT    800
Cla97C09G180070__812_     TTGAAGTTACATATGGTAAGTATCATGTGAGAACTTTAGCACTTCATGAGCTCGGACATGCACTTGGGCTGGCGCACAGCACCAATGAAGATGCTATCAT    800
Consensus                 ttgaagttacatatggtaagtatcatgtgagaacttt gcacttcatgagctcggacatgcacttgggctggcgcacagcaccaatgaagatgctatcat

Cla97C09G180070__1061_    GTTTCCCTCTCTATCTCCTAATGTTGTTAAGGATTTAGATATGGACGATGTTAATGGACTGTGGGAATTATATGATGGATTTGATGATGCTGAGTA        896
Cla97C09G180070__812_     GTTTCCCTCTCTATCTCCTAATGTTGTTAAGGATTTAGATATGGACGATGTTAATGGACTGTGGGAATTATATGATGGATTTGATGATGCTGAGTA        896
Consensus                 gtttccctctctatctcctaatgttgttaaggatttagatatggacgatgttaatggactgtgggaattatatgatggatttgatgatgctgagta
```

Figure S6G The result of CDS sequence alignment of *Cla97C09G180070* gene

```
Cla97C09G180070__1061_    MDFKSSSLQLFLLLLASIALFHPISTHDLDHIHKSSHFLFPQHLLGSRKDHNIEGIHSLRKNYNIIDTNGAHNNTFDHHLESAVKKYQKFFKLNESGILD    100
Cla97C09G180070__812_     MDFKSSSLQLFLLLLASIALFHPISTHDLDHIHKSSHFLFPQHLLGSRKDHNIEGIHSLRKNYNIIDTNGAHNNTFDHHLESAVKKYQKFFKLNESGILD    100
Consensus                 mdfkssslqlfllllasialfhpisthdldhihksshflfpqhllgsrkdhniegihslrknyniidtngahnntfdhhlesavkkyqkffklnesgild

Cla97C09G180070__1061_    VETLYQMSESRCSVPDIFEKDDNETSKLHIGSKYTFFPGRIKWASWKKYQLKYSFIRNFPEEFKESVSAAFMIWYERSRFNFTEVVENEDADIRISFEVG    200
Cla97C09G180070__812_     VETLYQMSESRCSVPDIFEKDDNETSKLHIGSKYTFFPGRIKWASWKKYQLKYSFIRNFPEEFKESVSAAFMIWYERSRFNFTEVVENEDADIRISFEVG    200
Consensus                 vetlyqmsesrcsvpdifekddnetsklhigskytffpgrikwaswkkyqlkysfirnfpeefkesvsaafmiwyersrfnftevvenedadirisfevg

Cla97C09G180070__1061_    NHGDLHPFTKEVLAHTFGPGDGRFHFNAEQSFSVEVTYGKYHVRTLALHELGHALGLAHSTNEDAIMFPSLSPNVVKDLDMDDVNGLWELYDGFDDA       297
Cla97C09G180070__812_     NHGDLHPFTKEVLAHTFGPGDGRFHFNAEQSFSVEVTYGKYHVRTLALHELGHALGLAHSTNEDAIMFPSLSPNVVKDLDMDDVNGLWELYDGFDDA       297
Consensus                 nhgdlhpftkevlahtfgpgdgrfhfnaeqsfsvevtygkyhvrtlalhelghalglahstnedaimfpslspnvvkdldmddvnglwelydgfdda
```

Figure S6G-1 The result of Cla97C09G180070 protein sequence alignment

```
Cla97C09G180100__1061_    ATGACTTTCAAGTTTCTTCGACTACTCTTTCTTGTTCTCGCCTCCATTGCACCTCACGTAATCTCCAAATACAATCGACCAATTGCATTTCCACAACATC    100
Cla97C09G180100__812_     ATGACTTTCAAGTTTCTTCAACTACTCTTTCTTGTTCTCGCCTCCATTGCACCTCACGTAATCTCCAAATACAATCGACCAATTGCATTTCCACAACATC    100
Consensus                 atgactttcaagtttcttc actactctttcttgttctcgcctccattgcacctcacgtaatctccaaatacaatcgaccaattgcatttccacaacatc

Cla97C09G180100__1061_    TTCAAGGATGTCGTAAAGGTGACATCGTAAAAGGAATCCACAACATTAGAACATATCTTCAACGTTATGGTTACTTATTACACAACACGAGTATCAATCC    200
Cla97C09G180100__812_     TTCAAGGATGTCGTAAAGGTGACATCGTAAAAGGAATCCACAACATTAGAACATATCTTCAACGTTATGGTTACTTATTACACAACACGAGTATCAATCC    200
Consensus                 ttcaaggatgtcgtaaaggtgacatcgtaaaaggaatccacaacattagaacatatcttcaacgttatggttacttattacacaacacgagtatcaatcc

Cla97C09G180100__1061_    CCACACTAATGAATTAACCGATGACACGTTCGATGGTGCCTTAGAATCCGCCATTAAATCATACCAAAAACGCTTCAACCTAAACACGACTGGGATTTTA    300
Cla97C09G180100__812_     CCACACTAATGAATTAACCGATGACACGTTCGATGGTGCCTTAGAATCCGCCATTAAATCATACCAAAAACGCTTCAACCTAAACACGACTGGGATTTTA    300
Consensus                 ccacactaatgaattaaccgatgacacgttcgatggtgccttagaatccgccattaaatcataccaaaaacgcttcaacctaaacacgactgggatttta

Cla97C09G180100__1061_    GACGAACAAACATTAGCTCAAATCTCCAAACCTCGATGTGGGGTACCGGATTTCTTCAACTCCAACCCGAACAAGAATCCTGAGGATGATCTCAAAATGT    400
Cla97C09G180100__812_     GACGAACAAACATTAGCTCAAATCTCCAAACCTCGATGTGGGGTACCGGATTTCTTCAACTCCAACCCGAACAAGAATCCTGAGGATGATCTCAAAATGT    400
Consensus                 gacgaacaaacattagctcaaatctccaaacctcgatgtggggtaccggatttcttcaactccaacccgaacaagaatcctgaggatgatctcaaaatgt

Cla97C09G180100__1061_    CATCTCATTACACTTTCTTCCCAGACAACCTAAGATGGCCAAATAACAAATTCAGTCTAACCTACACATTTACTAACAATTACCCATTAAACTTTGTACC    500
Cla97C09G180100__812_     CATCTCATTACACTTTCTTCCCAGACAACCTAAGATGGCCAAATAACAAATTCAGTCTAACCTACACATTTACTAACAATTACCCATTAAACTTTGTACC    500
Consensus                 catctcattacactttcttcccagacaacctaagatggccaaataacaaattcagtctaacctacacatttactaacaattacccattaaactttgtacc

Cla97C09G180100__1061_    GCCAGTGACTCGGGCTTTGGCTACATGGGCAGCTAACTCACAATTCACATTTTCAGAAGCTATGGAGGGACAAATGGCTGACATTAATATAAGCTTTCAA    600
Cla97C09G180100__812_     GCCAGTGACTCGGGCTTTGGCTACATGGGCAGCTAACTCACAATTCACATTTTCAGAAGCTATGGAGGGACAAATGGCTGACATTAATATAAGCTTTCAA    600
Consensus                 gccagtgactcgggctttggctacatgggcagctaactcacaattcacattttcagaagctatggagggacaaatggctgacattaatataagctttcaa

Cla97C09G180100__1061_    AGAGGGGAGCATGGAGATGAGAATCCTTTTGATGGACCTGGAGGAATTTTGGCTCATGCTTTTGCACCAACTGATGGGAGATTGCATTTTGATGGGGATG    700
Cla97C09G180100__812_     AGAGGGGAGCATGGAGATGAGAATCCTTTTGATGGACCTGGAGGAATTTTGGCTCATGCTTTTGCACCAACTGATGGGAGATTGCATTTTGATGGGGATG    700
Consensus                 agaggggagcatggagatgagaatccttttgatggacctggaggaattttggctcatgcttttgcaccaactgatgggagattgcattttgatggggatg

Cla97C09G180100__1061_    AGAGTTGGGCGGCGGGAGTGGTTGCTAATGAGTTAAATGTGGAGGCAGTGGCACTTCATGAGCTTGGACATGTTCTTGGGCTTGGCCATAGCTCCATTGA    800
Cla97C09G180100__812_     AGAGTTGGGCGGCGGGAGTGGTTGCTAATGAGTTAAATGTGGAGGCAGTGGCACTTCATGAGCTTGGACATGTTCTTGGGCTTGGCCATAGCTCCATTGA    800
Consensus                 agagttgggcggcgggagtggttgctaatgagttaaatgtggaggcagtggcacttcatgagcttggacatgttcttgggcttggccatagctccattga

Cla97C09G180100__1061_    ACAAGCGATCATGTGGCCCTATATTGAGGCTGGTTCTTCCAAGGGATTGGATGATGATGATATTGCTGGACTTCGGGCTTTGTATGCCTA            890
Cla97C09G180100__812_     ACAAGCGATCATGTGGCCCTATATTGAGGCTGGTTCTTCCAAGGGATTGGATGATGATGATATTGCTGGACTTCGGGCTTTGTATGCCTA            890
Consensus                 acaagcgatcatgtggccctatattgaggctggttcttccaagggattggatgatgatgatattgctggacttcgggctttgtatgccta
```

Figure S6H The result of CDS sequence alignment of *Cla97C09G180100* gene.

```
Cla97C09G180100__1061_    MTFKFLRLLFLVLASIAPHVISKYNRPIAFPQHLQGCRKGDIVKGIHNIRTYLQRYGYLLHNTSINPHTNELTDDTFDGALESAIKSYQKRFNLNTTGIL    100
Cla97C09G180100__812_     MTFKFLQLLFLVLASIAPHVISKYNRPIAFPQHLQGCRKGDIVKGIHNIRTYLQRYGYLLHNTSINPHTNELTDDTFDGALESAIKSYQKRFNLNTTGIL    100
Consensus                 mtfkfl llflvlasiaphviskynrpiafpqhlqgcrkgdivkgihnirtylqrygyllhntsinphtneltddtfdgalesaiksyqkrfnlnttgil

Cla97C09G180100__1061_    DEQTLAQISKPRCGVPDFFNSNPNKNPEDDLKMSSHYTFFPDNLRWPNNKFSLTYTFTNNYPLNFVPPVTRALATWAANSQFTFSEAMEGQMADINISFQ    200
Cla97C09G180100__812_     DEQTLAQISKPRCGVPDFFNSNPNKNPEDDLKMSSHYTFFPDNLRWPNNKFSLTYTFTNNYPLNFVPPVTRALATWAANSQFTFSEAMEGQMADINISFQ    200
Consensus                 deqtlaqiskprcgvpdffnsnpnknpeddlkmsshytffpdnlrwpnnkfsltytftnnyplnfvppvtralatwaansqftfseamegqmadinisfq

Cla97C09G180100__1061_    RGEHGDENPFDGPGGILAHAFAPTDGRLHFDGDESWAAGVVANELNVEAVALHELGHVLGLGHSSIEQAIMWPYIEAGSSKGLDDDDIAGLRALY           295
Cla97C09G180100__812_     RGEHGDENPFDGPGGILAHAFAPTDGRLHFDGDESWAAGVVANELNVEAVALHELGHVLGLGHSSIEQAIMWPYIEAGSSKGLDDDDIAGLRALY           295
Consensus                 rgehgdenpfdgpggilahafaptdgrlhfdgdeswaagvvanelnveavalhelghvlglghssieqaimwpyieagsskglddddiaglraly
```

Figure S6H-1 The result of Cla97C09G180100 protein sequence alignment

```
Cla97C09G180160__1061_   ATGGCGAAGAAGAAGGAAAAGAAGGTAAATGTGTCTGGTAAACCAAAGCATTCTCTGGACGTCAACCGAAGCGATGGGAACAAGAATTCACGTACTGCTG   100
Cla97C09G180160__812_    ATGGCGAAGAAGAAGGAAAAGAAGGTAAATGTGTCTGGTAAACCAAAGCATTCTCTGGACGTCAACCGAAGCGATGGGAACAAGAATTCACGTACTGCTG   100
Consensus                atggcgaagaagaaggaaaagaaggtaaatgtgtctggtaaaccaaagcattctctggacgtcaaccgaagcgatgggaacaagaattcacgtactgctg

Cla97C09G180160__1061_   CCACTGTGCGGCGTCTCAAGATGTATAATACGAGGCCAAAACGTGATCGGAAAGGGAAGGTGTTGAAGCATGAGCTTCAGTCGAAGGAGTTACCTGACAC   200
Cla97C09G180160__812_    CCACTGTGCGGCGTCTCAAGATGTATAATACGAGGCCAAAACGTGATCGGAAAGGGAAGGTGTTGAAGCATGAGCTTCAGTCGAAGGAGTTACCTGACAC   200
Consensus                ccactgtgcggcgtctcaagatgtataatacgaggccaaaacgtgatcggaaagggaaggtgttgaagcatgagcttcagtcgaaggagttacctgacac

Cla97C09G180160__1061_   ACGAATTCAACCTGATCGCCGTTGGTTCGGGAATACCCGAGTTGTAAACCAGAAAGAGCTTGAAATTTTTCGTGAAGAGCTAGAAAAACGGATGTCAAAT   300
Cla97C09G180160__812_    ACGAATTCAACCTGATCGCCGTTGGTTCGGGAATACCCGAGTTGTAAACCAGAAAGAGCTTGAAATTTTTCGTGAAGAGCTAGAAAAACGGATGTCAAAT   300
Consensus                acgaattcaacctgatcgccgttggttcgggaatacccgagttgtaaaccagaaagagcttgaaatttttcgtgaagagctagaaaaacggatgtcaaat

Cla97C09G180160__1061_   AACTATAATGTGATTTTGAAGGAAAGGAAGCTGCCCCTTTCCCTGTTGAATGATCATCAAAAGCAATCCAGAGTCCATCTTCTCGATACAGAACCTTTTC   400
Cla97C09G180160__812_    AACTATAATGTGATTTTGAAGGAAAGGAAGCTGCCCCTTTCCCTGTTGAATGATCATCAAAAGCAATCCAGAGTCCATCTTCTCGATACAGAACCTTTTC   400
Consensus                aactataatgtgattttgaaggaaaggaagctgcccctttccctgttgaatgatcatcaaaagcaatccagagtccatcttctcgatacagaaccttttc

Cla97C09G180160__1061_   AGGATGCATTTGGGCCAAAGGGGAAGAGAAAGCGACCAAAGCTTTTGGCTGCTGACTATGAGTCACTACTTAAGAAAGCTGACAAGTCCCATGATGACTT   500
Cla97C09G180160__812_    AGGATGCATTTGGGCCAAAGGGGAAGAGAAAGCGACCAAAGCTTTTGGCTGCTGACTATGAGTCACTACTTAAGAAAGCTGACAAGTCCCATGATGACTT   500
Consensus                aggatgcatttgggccaaaggggaagagaaagcgaccaaagcttttggctgctgactatgagtcactacttaagaaagctgacaagtcccatgatgactt

Cla97C09G180160__1061_   TGAGGAAAAGTATGCTGAAAATGCTACTGTAGAGGGAAGCGAGGAAGATGGTTTTAGAGACCTAGTTCGACACACTATGTTTGAGAAGGGTCAAAGTAAA   600
Cla97C09G180160__812_    TGAGGAAAAGTATGCTGAAAATGCTACTGTAGAGGGAAGCGAGGAAGATGGTTTTAGAGACCTAGTTCGACACACTATGTTTGAGAAGGGTCAAAGTAAA   600
Consensus                tgaggaaaagtatgctgaaaatgctactgtagagggaagcgaggaagatggttttagagacctagttcgacacactatgtttgagaagggtcaaagtaaa

Cla97C09G180160__1061_   CGTATATGGGGTGAGCTTTACAAAGTGATCGATTCTTCAGATGTTGTTGTCCAGGTTCTAGATGCAAGAGATCCACAAGGAACAAGATGTTACCATTTAG   700
Cla97C09G180160__812_    CGTATATGGGGTGAGCTTTACAAAGTGATCGATTCTTCAGATGTTGTTGTCCAGGTTCTAGATGCAAGAGATCCACAAGGAACAAGATGTTACCATTTAG   700
Consensus                cgtatatggggtgagctttacaaagtgatcgattcttcagatgttgttgtccaggttctagatgcaagagatccacaaggaacaagatgttaccatttag

Cla97C09G180160__1061_   AGAGACATTTGAAAGAGCATTGCAAACATAAACACGTGGTTCTCTTGTTAAATAAGTGCGATTTGATTCCTGCTTGGGCAACAAAGGGATGGCTTAGAGT   800
Cla97C09G180160__812_    AGAGACATTTGAAAGAGCATTGCAAACATAAACACGTGGTTCTCTTGTTAAATAAGTGCGATTTGATTCCTGCTTGGGCAACAAAGGGATGGCTTAGAGT   800
Consensus                agagacatttgaaagagcattgcaaacataaacacgtggttctcttgttaaataagtgcgatttgattcctgcttgggcaacaaagggatggcttagagt

Cla97C09G180160__1061_   GTTATCAAAAGAATATCCAACTCTAGCATTTCATGCAAGCATCAACAAATCCTTTGGAAAGGGTTCTCTCCTGTCCGTGCTGAGACAATTTGCTCGATTA   900
Cla97C09G180160__812_    GTTATCAAAAGAATATCCAACTCTAGCATTTCATGCAAGCATCAACAAATCCTTTGGAAAGGGTTCTCTCCTGTCCGTGCTGAGACAATTTGCTCGATTA   900
Consensus                gttatcaaaagaatatccaactctagcatttcatgcaagcatcaacaaatcctttggaaagggttctctcctgtccgtgctgagacaatttgctcgatta

Cla97C09G180160__1061_   AAAAGTGACAAGCAAGCTATCTCTGTTGGATTTGTTGGGTATCCCAATGTTGGAAAGTCATCTGTAATTAACACTCTACGGACTAAGAATGTGTGCAAAG   1000
Cla97C09G180160__812_    AAATGTGACAAGCAAGCTATCTCTGTTGGATTTGTTGGGTATCCCAATGTTGGAAAGTCATCTGTAATTAACACTCTACGGACTAAGAATGTGTGCAAAG   1000
Consensus                aaa gtgacaagcaagctatctctgttggatttgttgggtatcccaatgttggaaagtcatctgtaattaacactctacggactaagaatgtgtgcaaag

Cla97C09G180160__1061_   TTGCACCTATTCCAGGGGAAACTAAAGTTTGGCAATATATAACTCTCACAAAGAGGATTTTCCTGATTGATTGCCCGGGAGTTGTTTACCAGAATAGTGA   1100
Cla97C09G180160__812_    TTGCACCTATTCCAGGGGAAACTAAAGTTTGGCAATATATAACTCTCACAAAGAGGATTTTCCTGATTGATTGCCCGGGAGTTGTTTACCAGAATAGTGA   1100
Consensus                ttgcacctattccaggggaaactaaagtttggcaatatataactctcacaaagaggattttcctgattgattgcccgggagttgtttaccagaatagtga

Cla97C09G180160__1061_   CACTGAAACTGATATCGTGCTTAAGGGCGTGGTACGAGTTACAAATTTGGAGGATGCAGCCGAACATATTGGAGAAGTTTTGAAGCGTGTGAAGAAGGAA   1200
Cla97C09G180160__812_    CACTGAAACTGATATCGTGCTTAAGGGCGTGGTACGAGTTACAAATTTGGAGGATGCAGCCGAACATATTGGAGAAGTTTTGAAGCGTGTGAAGAAGGAA   1200
Consensus                cactgaaactgatatcgtgcttaagggcgtggtacgagttacaaatttggaggatgcagccgaacatattggagaagttttgaagcgtgtgaagaaggaa

Cla97C09G180160__1061_   CACCTTGAAAGAGCATACAGGATAAAAAATTGGGAGGACGATAATGACTTTTTAGTTCAGCTTTGCAAATTGACAGGGAAGCTCCTAAGGGGTGGCGAAC   1300
Cla97C09G180160__812_    CACCTTGAAAGAGCATACAGGATAAAAAATTGGGAGGACGATAATGACTTTTTAGTTCAGCTTTGCAAATTGACAGGGAAGCTCCTAAGGGGTGGCGAAC   1300
Consensus                caccttgaaagagcatacaggataaaaaattgggaggacgataatgactttttagttcagctttgcaaattgacagggaagctcctaaggggtggcgaac

Cla97C09G180160__1061_   CTGACTTGACCACTGCAGCAAAAATGGTCCTCCATGACTGGCAGAGGGGCAGACTTCCCTTTTTTGTTCCGCCACCTCGAGTAGAAGATGAATCAGAAGA   1400
Cla97C09G180160__812_    CTGACTTGACCACTGCAGCAAAAATGGTCCTCCATGACTGGCAGAGGGGCAGACTTCCCTTTTTTGTTCCGCCACCTCGAGTAGAAGATGAATCAGAAGA   1400
Consensus                ctgacttgaccactgcagcaaaaatggtcctccatgactggcagaggggcagacttcccttttttgttccgccacctcgagtagaagatgaatcagaaga

Cla97C09G180160__1061_   ACCCAACTATGGTGTTGATGATGACTCAGGTGTCGAAAGCAATCAAGCTGCAGCCGCTTTCAAAGCCATTGCAAGTGTGATATCATCTCAGCAGCAAAGA   1500
Cla97C09G180160__812_    ACCCAACTATGGTGTTGATGATGACTCAGGTGTCGAAAGCAATCAAGCTGCAGCCGCTTTCAAAGCCATTGCAAGTGTGATATCATCTCAGCAGCAAAGA   1500
Consensus                acccaactatggtgttgatgatgactcaggtgtcgaaagcaatcaagctgcagccgctttcaaagccattgcaagtgtgatatcatctcagcagcaaaga

Cla97C09G180160__1061_   AGTGTGCCTGTTCAAAGGGATCTGTTTAGTGATAATGAATTGAATGGCGAGGCATCCGATCAGATTCTAGTCTCTGAGGATGAGTTACAGGCTCATCCTT   1600
Cla97C09G180160__812_    AGTGTGCCTGTTCAAAGGGATCTGTTTAGTGATAATGAATTGAATGGCGAGGCATCCGATCAGATTCTAGTCTCTGAGGATGAGTTACAGGCTCATCCTT   1600
Consensus                agtgtgcctgttcaaagggatctgtttagtgataatgaattgaatggcgaggcatccgatcagattctagtctctgaggatgagttacaggctcatcctt

Cla97C09G180160__1061_   CTGAGACTGAGGGAAAAACATCAGGAGACGAGGATGACGATGATGAAGATGAGCGTCCGATCGCAGGCTG                                 1670
Cla97C09G180160__812_    CTGAGACTGAGGGAAAAACATCAGGAGACGAGGATGACGATGATGAAGATGAGCGTCCGATCGCAGGCTG                                 1670
Consensus                ctgagactgagggaaaaacatcaggagacgaggatgacgatgatgaagatgagcgtccgatcgcaggctg
```

Figure S6I The result of CDS sequence alignment of *Cla97C09G180160* gene

```
Cla97C09G180160__1061_   MAKKKEKKVNVSGKPKHSLDVNRSDGNKNSRTAATVRRLKMYNTRPKRDRKGKVLKHELQSKELPDTRIQPDRRWFGNTRVVNQKELEIFREELEKRMSN   100
Cla97C09G180160__812_    MAKKKEKKVNVSGKPKHSLDVNRSDGNKNSRTAATVRRLKMYNTRPKRDRKGKVLKHELQSKELPDTRIQPDRRWFGNTRVVNQKELEIFREELEKRMSN   100
Consensus                makkkekkvnvsgkpkhsldvnrsdgnknsrtaatvrrlkmyntrpkrdrkgkvlkhelqskelpdtriqpdrrwfgntrvvnqkeleifreelekrmsn

Cla97C09G180160__1061_   NYNVILKERKLPLSLLNDHQKQSRVHLLDTEPFQDAFGPKGKRKRPKLLAADYESLLKKADKSHDDFEEKYAENATVEGSEEDGFRDLVRHTMFEKGQSK   200
Cla97C09G180160__812_    NYNVILKERKLPLSLLNDHQKQSRVHLLDTEPFQDAFGPKGKRKRPKLLAADYESLLKKADKSHDDFEEKYAENATVEGSEEDGFRDLVRHTMFEKGQSK   200
Consensus                nynvilkerklplsllndhqkqsrvhlldtepfqdafgpkgkrkrpkllaadyesllkkadkshddfeekyaenatvegseedgfrdlvrhtmfekgqsk

Cla97C09G180160__1061_   RIWGELYKVIDSSDVVVQVLDARDPQGTRCYHLERHLKEHCKHKHVVLLLNKCDLIPAWATKGWLRVLSKEYPTLAFHASINKSFGKGSLLSVLRQFARL   300
Cla97C09G180160__812_    RIWGELYKVIDSSDVVVQVLDARDPQGTRCYHLERHLKEHCKHKHVVLLLNKCDLIPAWATKGWLRVLSKEYPTLAFHASINKSFGKGSLLSVLRQFARL   300
Consensus                riwgelykvidssdvvvqvldardpqgtrcyhlerhlkehckhkhvvlllnkcdlipawatkgwlrvlskeyptlafhasinksfgkgsllsvlrqfarl

Cla97C09G180160__1061_   KSDKQAISVGFVGYPNVGKSSVINTLRTKNVCKVAPIPGETKVWQYITLTKRIFLIDCPGVVYQNSDTETDIVLKGVVRVTNLEDAAEHIGEVLKRVKKE   400
Cla97C09G180160__812_    KCDKQAISVGFVGYPNVGKSSVINTLRTKNVCKVAPIPGETKVWQYITLTKRIFLIDCPGVVYQNSDTETDIVLKGVVRVTNLEDAAEHIGEVLKRVKKE   400
Consensus                k dkqaisvgfvgypnvgkssvintlrtknvckvapipgetkvwqyitltkriflidcpgvvyqnsdtetdivlkgvvrvtnledaaehigevlkrvkke

Cla97C09G180160__1061_   HLERAYRIKNWEDDNDFLVQLCKLTGKLLRGGEPDLTTAAKMVLHDWQRGRLPFFVPPPRVEDESEEPNYGVDDDSGVESNQAAAAFKAIASVISSQQQR   500
Cla97C09G180160__812_    HLERAYRIKNWEDDNDFLVQLCKLTGKLLRGGEPDLTTAAKMVLHDWQRGRLPFFVPPPRVEDESEEPNYGVDDDSGVESNQAAAAFKAIASVISSQQQR   500
Consensus                hlerayriknweddndflvqlckltgkllrggepdlttaakmvlhdwqrgrlpffvppprvedeseepnygvdddsgvesnqaaaafkaiasvissqqqr

Cla97C09G180160__1061_   SVPVQRDLFSDNELNGEASDQILVSEDELQAHPSETEGKTSGDEDDDDEDERPIA                                                555
Cla97C09G180160__812_    SVPVQRDLFSDNELNGEASDQILVSEDELQAHPSETEGKTSGDEDDDDEDERPIA                                                555
Consensus                svpvqrdlfsdnelngeasdqilvsedelqahpsetegktsgdeddddederpia
```

Figure S6I-1 The result of Cla97C09G180160 protein sequence alignment

```
Cla97C09G180170__1061_    ATGGCTGAAAATGAACAGGAGATGCGATCTTTGGCTTTGACGCCCACTTGGTCTGTTGCTTCTGTGCTGACTATTTTCGTTGCAGTCTCTTTGCTTGTTG    100
Cla97C09G180170__812_     ATGGCTGAAAATGAACAGGAGATGCGATCTTTGGCTTTGACGCCCACTTGGTCTGTTGCTTCTGTGCTGACTATTTTCGTTGCAGTCTCTTTGCTTGTTG    100
Consensus                 atggctgaaaatgaacaggagatgcgatctttggctttgacgcccacttggtctgttgcttctgtgctgactattttcgttgcagtctctttgcttgttg

Cla97C09G180170__1061_    AGCGGTCCATTCACCGGTTAAGCACTTGGTTGGGGAAAACTAACCGAAAGCCACTCTTTGAGGCAGTGGAGAAAATGAAAGAAGAGTTGATGCTGCTTGG    200
Cla97C09G180170__812_     AGCGGTCCATTCACCGGTTAAGCACTTGGTTGGGGAAAACTAACCGAAAGCCACTCTTTGAGGCAGTGGAGAAAATGAAAGAAGAGTTGATGCTGCTTGG    200
Consensus                 agcggtccattcaccggttaagcacttggttggggaaaactaaccgaaagccactctttgaggcagtggagaaaatgaaagaagagttgatgctgcttgg

Cla97C09G180170__1061_    ATTTATTTCTCTCCTTTTAACTGCTACATCAAGCTCAATAGCAAATATCTGCATCCCATCAAAGTTCTACAGTACCCCTTTTACTCCATGCACCAAAGCT    300
Cla97C09G180170__812_     ATTTATTTCTCTCCTTTTAACTGCTACATCAAGCTCAATAGCAAATATCTGCATCCCATCAAAGTTCTACAGTACCCCTTTTACTCCATGCACCAAAGCT    300
Consensus                 atttatttctctccttttaactgctacatcaagctcaatagcaaatatctgcatcccatcaaagttctacagtaccccttttactccatgcaccaaagct

Cla97C09G180170__1061_    GAGGTTGATGAACAGACAGATGACAGTTCATCTGAGGAACGGAAACTCTATACAGTTTCTGTATTACCCCATTTGTTTAGGCGGATGCTTAATGCGAATA    400
Cla97C09G180170__812_     GAGGTTGATGAACAGACAGATGACAGTTCATCTGAGGAACGGAAACTCTATACAGTTTCTATATTACCCCATTTGTTTAGGCGGATGCTTAATGCGAATA    400
Consensus                 gaggttgatgaacagacagatgacagttcatctgaggaacggaaactctatacagtttct tattaccccatttgtttaggcggatgcttaatgcgaata

Cla97C09G180170__1061_    GGAAAACCTGCAAAAAGGGTTATGAGCCGTTTGTTTCATATGAGGGTCTTGAGCAATTGCATCGCTTTATCTTTATAATGGCAGTAACTCATATATCTTA    500
Cla97C09G180170__812_     GGAAAACCTGCAAAAAGGGTTATGAGCCGTTTGTTTCATATGAGGGTCTTGAGCAATTGCATCGCTTTATCTTTATAATGGCAGTAACTCATATATCTTA    500
Consensus                 ggaaaacctgcaaaaagggttatgagccgtttgtttcatatgagggtcttgagcaattgcatcgctttatctttataatggcagtaactcatatatctta

Cla97C09G180170__1061_    TAGCTGCTTAACAATGTTACTGGCTATTGTGAAGATTCACAGATGGAGAGTCTGGGAGGATGAAGCCCACATGGACAGACATGATTCACTAAATGATATC    600
Cla97C09G180170__812_     TAGCTGCTTAACAATGTTACTGGCTATTGTGAAGATTCACAGATGGAGAGTCTGGGAGGATGAAGCCCACATGGACAGACATGATTCACTAAATGATATC    600
Consensus                 tagctgcttaacaatgttactggctattgtgaagattcacagatggagagtctgggaggatgaagcccacatggacagacatgattcactaaatgatatc

Cla97C09G180170__1061_    ACAAGAGAAATGACACTGCGGAGGCAATCAACGTTTGTTCGATATCACACTTCAAATCCTATGACGAGGAACAGTTTTCTAATCTGGGTGACATGTTTTT    700
Cla97C09G180170__812_     ACAAGAGAAATGACACTGCGGAGGCAATCAACGTTTGTTCGATATCACACTTCAAATCCTATGACGAGGAACAGTTTTCTAATCTGGGTGACATGTTTTT    700
Consensus                 acaagagaaatgacactgcggaggcaatcaacgtttgttcgatatcacacttcaaatcctatgacgaggaacagttttctaatctgggtgacatgttttt

Cla97C09G180170__1061_    TCCGGCAATTTGGAAATTCTGTAGTTCGTGCTGACTACCTCACACTCCGCAAAGGCTTCATCATGAATCACCACCTCCCCTTGACATATGATTTCCACAG    800
Cla97C09G180170__812_     TCCGGCAATTTGGAAATTCTGTAGTTCGTGCTGACTACCTCACACTCCGCAAAGGCTTCATCATGAATCACCACCTCCCCTTGACATATGATTTCCACAG    800
Consensus                 tccggcaatttggaaattctgtagttcgtgctgactacctcacactccgcaaaggcttcatcatgaatcaccacctccccttgacatatgatttccacag

Cla97C09G180170__1061_    CTACATGATTCGCTCCATGGAAGAAGAATTCCAAAGGATAGTAGGTGTGAGTGGTCCATTATGGGGATTCGTTGTTGCTTTCATGCTGTTTAATGTAAAA    900
Cla97C09G180170__812_     CTACATGATTCGCTCCATGGAAGAAGAATTCCAAAGGATAGTAGGTGTGAGTGGTCCATTATGGGGATTCGTTGTTGCTTTCATGCTGTTTAATGTAAAA    900
Consensus                 ctacatgattcgctccatggaagaagaattccaaaggatagtaggtgtgagtggtccattatggggattcgttgttgctttcatgctgtttaatgtaaaa

Cla97C09G180170__1061_    GGCTCTAATCTGTATTTCTGGATAGCAAGCATTCCAATTGCTCTTGTTCTGTTGGTGGGCACGAAGCTGCAGCATGTCATTGCAACATTAGCATTGGAAA    1000
Cla97C09G180170__812_     GGCTCTAATCTGTATTTCTGGATAGCAAGCATTCCAATTGCTCTTGTTCTGTTGGTGGGCACGAAGCTGCAGCATGTCATTGCAACATTAGCATTGGAAA    1000
Consensus                 ggctctaatctgtatttctggatagcaagcattccaattgctcttgttctgttggtgggcacgaagctgcagcatgtcattgcaacattagcattggaaa

Cla97C09G180170__1061_    GTGCTGGTATAACTGGTTCATTTTCGGGTTCAAAGCTAAAGCCAAGAGATGATCTTTTTTGGTTTAAGAAGCCAGAGCTCCTCTTGTCCTTGATCCACTT    1100
Cla97C09G180170__812_     GTGCTGGTATAACTGGTTCATTTTCGGGTTCAAAGCTAAAGCCAAGAGATGATCTTTTTTGGTTTAAGAAGCCAGAGCTCCTCTTGTCCTTGATCCACTT    1100
Consensus                 gtgctggtataactggttcattttcgggttcaaagctaaagccaagagatgatcttttttggtttaagaagccagagctcctcttgtccttgatccactt

Cla97C09G180170__1061_    TATCCTTTTCCAGAACGCATTCGAGTTGGCATCATTCTTCTGGTTCTGGTGGCAATTCGGATATAATTCTTGCTTCATTAGGAATCATATGCTTGTCTAT    1200
Cla97C09G180170__812_     TATCCTTTTCCAGAACGCATTCGAGTTGGCATCATTCTTCTGGTTCTGGTGGCAATTCGGATATAATTCTTGCTTCATTAGGAATCATATGCTTGTCTAT    1200
Consensus                 tatccttttccagaacgcattcgagttggcatcattcttctggttctggtggcaattcggatataattcttgcttcattaggaatcatatgcttgtctat

Cla97C09G180170__1061_    GCAAGACTTCTTTTGGGATTCGCTGGGCAGTTCCTTTGCAGCTACAGCACCTTGCCCTTATATGCTTTGGTAACTCAGATGGGAACAAACTATAAAGCTG    1300
Cla97C09G180170__812_     GCAAGGCTTCTTTTGGGATTCGCTGGGCAGTTCCTTTGCAGCTACAGCACCTTGCCCTTATATGCTTTGGTAACTCAGATGGGAACAAACTATAAAGCTG    1300
Consensus                 gcaag cttcttttgggattcgctgggcagttcctttgcagctacagcaccttgcccttatatgctttggtaactcagatgggaacaaactataaagctg

Cla97C09G180170__1061_    CATTAATTCCACAAAGAATAAGGGAAACAATTCATGGATGGGGGAAGGCAGCAAGGAGGAAAAGAAGGCTTCGCATGTTTGCAGATGACACCACAATTCA    1400
Cla97C09G180170__812_     CATTAATTCCACAAAGAATAAGGGAAACAATTCATGGATGGGGGAAGGCAGCAAGGAGGAAAAGAAGGCTTCGCATGTTTGCAGATGACACCACAATTCA    1400
Consensus                 cattaattccacaaagaataagggaaacaattcatggatgggggaaggcagcaaggaggaaaagaaggcttcgcatgtttgcagatgacaccacaattca

Cla97C09G180170__1061_    CACCGAAACAAGCACTGTGTTGTCACTTGAGGATGATGACCGTCGGCTTATCGATGATACTTCTGAAACTACTGCTGACTATACGGCAATCGAACTACAG    1500
Cla97C09G180170__812_     CACCGAAACAAGCACTGTGTTGTCACTTGAGGATGATGACCGTCGGCTTATCGATGATACTTCTGAAACTACTGCTGACTATACGGCAATCGAACTACAG    1500
Consensus                 caccgaaacaagcactgtgttgtcacttgaggatgatgaccgtcggcttatcgatgatacttctgaaactactgctgactatacggcaatcgaactacag

Cla97C09G180170__1061_    CTGACTACCGTACAAGATGAACCTGACTCTGTTACTAATGAACGACCAAGCAGGGCTAGGACGCCTCTTCTACAACCCTCTACATCTCTTTCTTCTGCAG    1600
Cla97C09G180170__812_     CTGACTACCGTACAAGATGAACCTGACTCTGTTACTAATGAACGACCAAGCAGGGCTAGGACGCCTCTTCTACAACCCTCTACATCTCTTTCTTCTGCAG    1600
Consensus                 ctgactaccgtacaagatgaacctgactctgttactaatgaacgaccaagcagggctaggacgcctcttctacaaccctctacatctctttcttctgcag

Cla97C09G180170__1061_    TTGATCATAAGTTTGAGGTGGAAAACTTTATGAGAAGCTTTTCTATGCCAGTCAAAAGATA                                           1661
Cla97C09G180170__812_     TTGATCATAAGTTTGAGGTGGAAAACTTTATGAGAAGCTTTTCTATGCCAGTCAAAAGATA                                           1661
Consensus                 ttgatcataagtttgaggtggaaaactttatgagaagcttttctatgccagtcaaaagata
```

Figure S6J The result of CDS sequence alignment of *Cla97C09G180170* gene

```
Cla97C09G180170__1061_    MAENEQEMRSLALTPTWSVASVLTIFVAVSLLVERSIHRLSTWLGKTNRKPLFEAVEKMKEELMLLGFISLLLTATSSSIANICIPSKFYSTPFTPCTKA    100
Cla97C09G180170__812_     MAENEQEMRSLALTPTWSVASVLTIFVAVSLLVERSIHRLSTWLGKTNRKPLFEAVEKMKEELMLLGFISLLLTATSSSIANICIPSKFYSTPFTPCTKA    100
Consensus                 maeneqemrslaltptwsvasvltifvavsllversihrlstwlgktnrkplfeavekmkeelmllgfisllltatsssianicipskfystpftpctka

Cla97C09G180170__1061_    EVDEQTDDSSSEERKLYTVSVLPHLFRRMLNANRKTCKKGYEPFVSYEGLEQLHRFIFIMAVTHISYSCLTMLLAIVKIHRWRVWEDEAHMDRHDSLNDI    200
Cla97C09G180170__812_     EVDEQTDDSSSEERKLYTVSILPHLFRRMLNANRKTCKKGYEPFVSYEGLEQLHRFIFIMAVTHISYSCLTMLLAIVKIHRWRVWEDEAHMDRHDSLNDI    200
Consensus                 evdeqtddssseerklytvs lphlfrrmlnanrktckkgyepfvsyegleqlhrfifimavthisyscltmllaivkihrwrvwedeahmdrhdslndi

Cla97C09G180170__1061_    TREMTLRRQSTFVRYHTSNPMTRNSFLIWVTCFFRQFGNSVVRADYLTLRKGFIMNHHLPLTYDFHSYMIRSMEEEFQRIVGVSGPLWGFVVAFMLFNVK    300
Cla97C09G180170__812_     TREMTLRRQSTFVRYHTSNPMTRNSFLIWVTCFFRQFGNSVVRADYLTLRKGFIMNHHLPLTYDFHSYMIRSMEEEFQRIVGVSGPLWGFVVAFMLFNVK    300
Consensus                 tremtlrrqstfvryhtsnpmtrnsfliwvtcffrqfgnsvvradyltlrkgfimnhhlpltydfhsymirsmeeefqrivgvsgplwgfvvafmlfnvk

Cla97C09G180170__1061_    GSNLYFWIASIPIALVLLVGTKLQHVIATLALESAGITGSFSGSKLKPRDDLFWFKKPELLLSLIHFILFQNAFELASFFWFWWQFGYNSCFIRNHMLVY    400
Cla97C09G180170__812_     GSNLYFWIASIPIALVLLVGTKLQHVIATLALESAGITGSFSGSKLKPRDDLFWFKKPELLLSLIHFILFQNAFELASFFWFWWQFGYNSCFIRNHMLVY    400
Consensus                 gsnlyfwiasipialvllvgtklqhviatlalesagitgsfsgsklkprddlfwfkkpelllslihfilfqnafelasffwfwwqfgynscfirnhmlvy

Cla97C09G180170__1061_    ARLLLGFAGQFLCSYSTLPLYALVTQMGTNYKAALIPQRIRETIHGWGKAARRKRRLRMFADDTTIHTETSTVLSLEDDDRRLIDDTSETTADYTAIELQ    500
Cla97C09G180170__812_     ARLLLGFAGQFLCSYSTLPLYALVTQMGTNYKAALIPQRIRETIHGWGKAARRKRRLRMFADDTTIHTETSTVLSLEDDDRRLIDDTSETTADYTAIELQ    500
Consensus                 arlllgfagqflcsystlplyalvtqmgtnykaalipqriretihgwgkaarrkrrlrmfaddttihtetstvlsledddrrliddtsettadytaielq

Cla97C09G180170__1061_    LTTVQDEPDSVTNERPSRARTPLLQPSTSLSSAVDHKFEVENFMRSFSMPVK                                                    552
Cla97C09G180170__812_     LTTVQDEPDSVTNERPSRARTPLLQPSTSLSSAVDHKFEVENFMRSFSMPVK                                                    552
Consensus                 lttvqdepdsvtnerpsrartpllqpstslssavdhkfevenfmrsfsmpvk
```

Figure S6J-1 The result of Cla97C09G180170 protein sequence alignment
