# Supplementary figures and images for: Molecular Mapping of Putative Genomic Regions Controlling Fruit and Seed Morphology of Watermelon
Source: Int J Mol Sci. 2023 Oct 30;24(21):15755. doi: 10.3390/ijms242115755 (PMC10650541; doi:10.3390/ijms242115755)

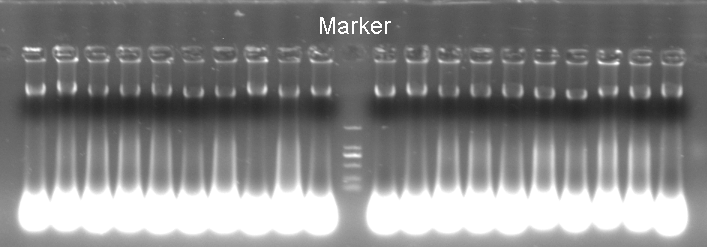

Supplement: Supplementary file 1 [file ijms-24-15755-s001.zip › Supplementary Figure S1.jpg]

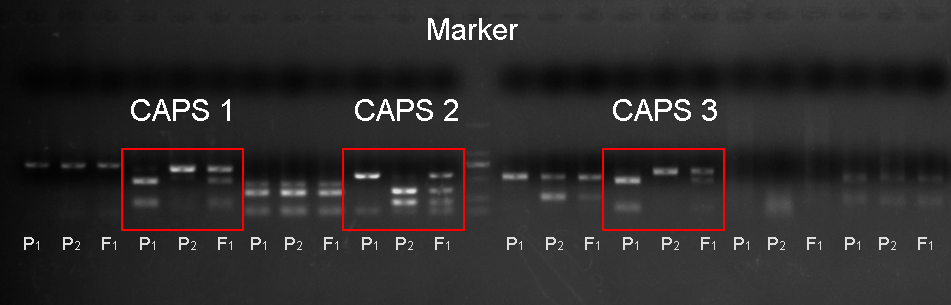

Supplement: Supplementary file 1 [file ijms-24-15755-s001.zip › Supplementary Figure S2.jpg]

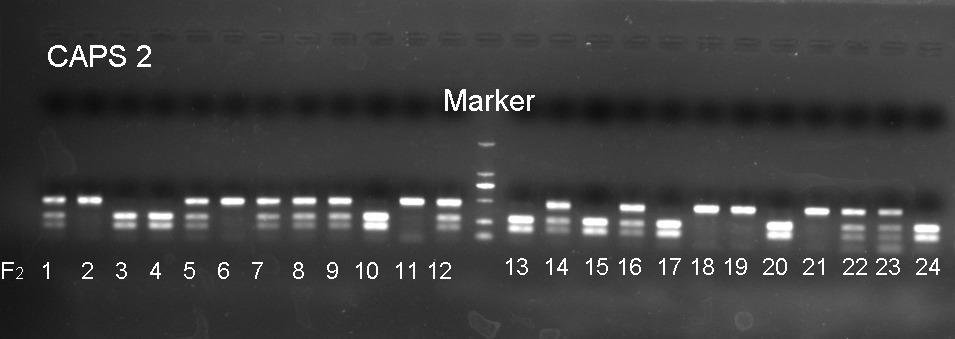

Supplement: Supplementary file 1 [file ijms-24-15755-s001.zip › Supplementary Figure S3.jpg]

Supplementary Figure S4A

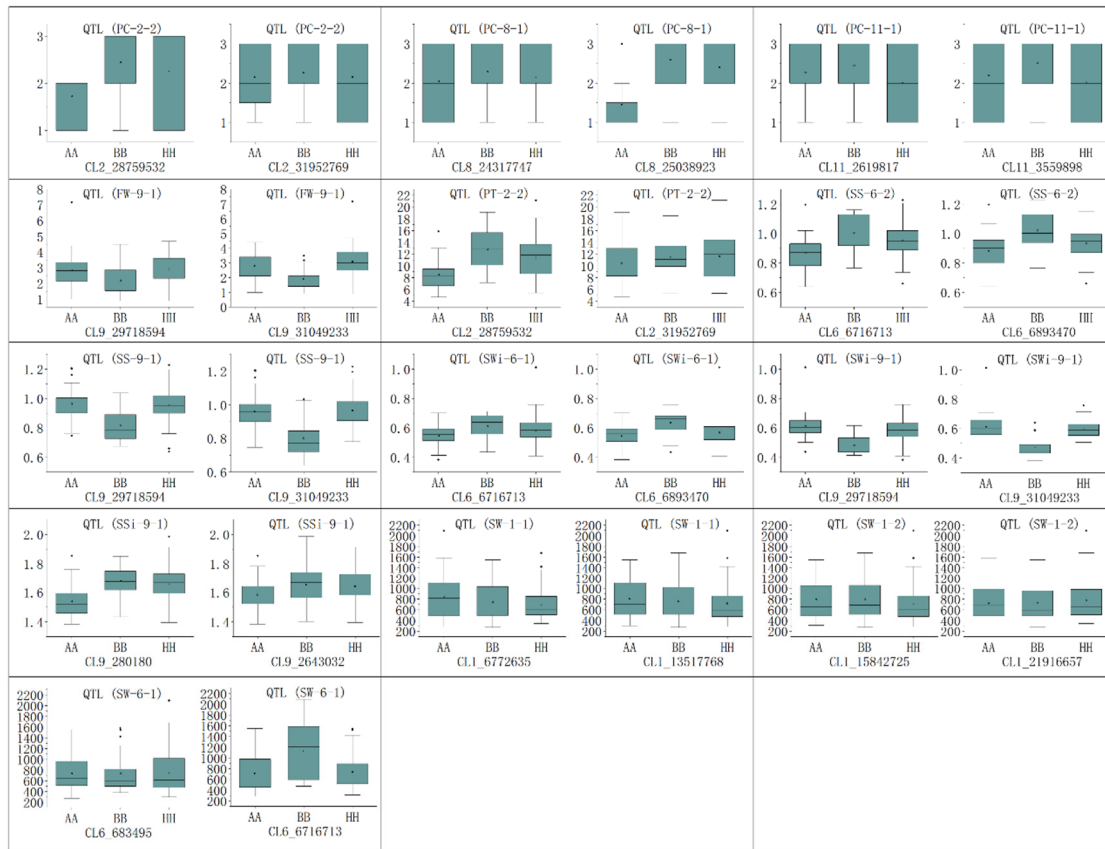

Supplementary Figure S4B

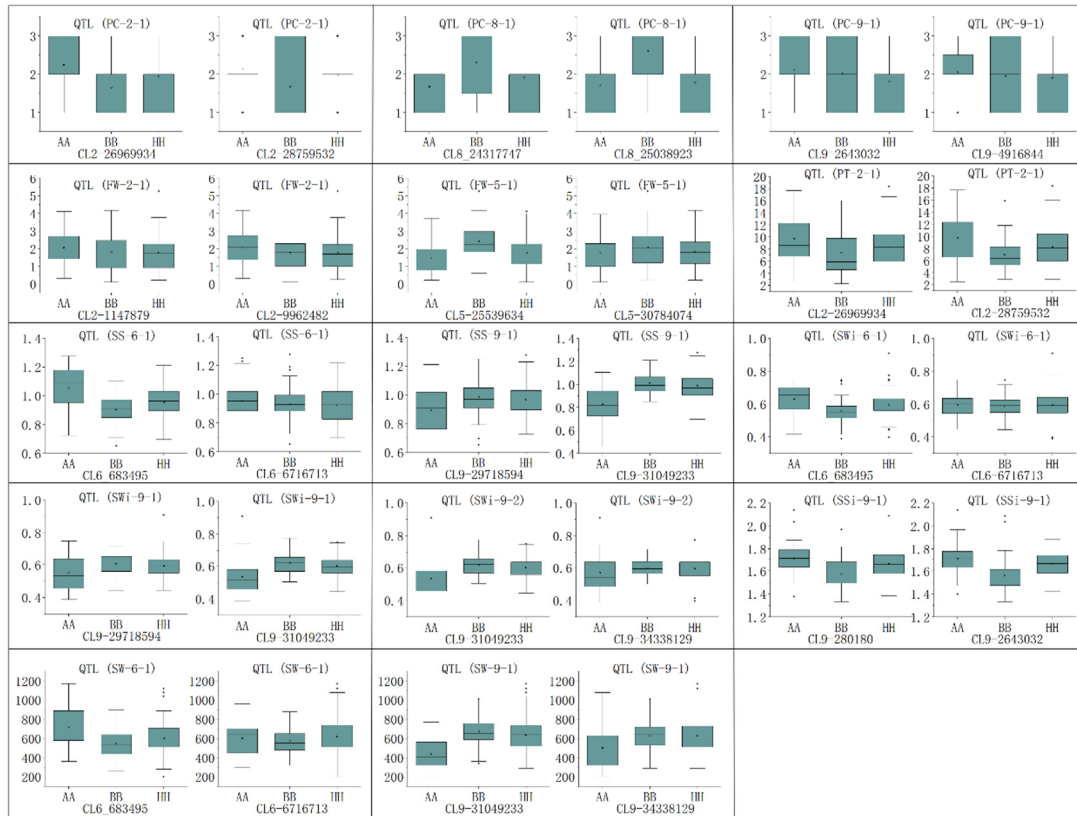

Supplement: Supplementary file 1 [file ijms-24-15755-s001.zip › Supplementary Figure S4.pdf]

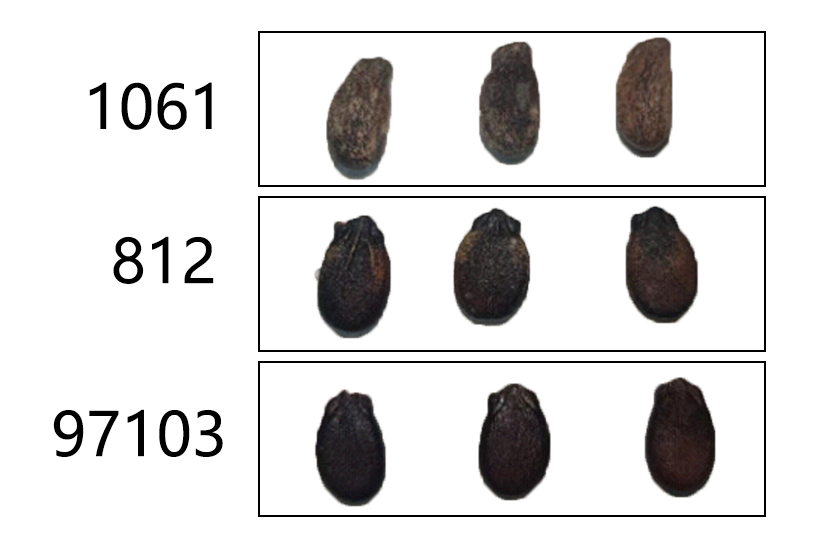

Supplement: Supplementary file 1 [file ijms-24-15755-s001.zip › Supplementary Figure S5.jpg]
